# Resampling to Address the Winner's Curse in Genetic Association Analysis of Time to Event

**DOI:** 10.1002/gepi.21920

**Published:** 2015-09-28

**Authors:** Julia G. Poirier, Laura L. Faye, Apostolos Dimitromanolakis, Andrew D. Paterson, Lei Sun, Shelley B. Bull

**Affiliations:** ^1^ Lunenfeld‐Tanenbaum Research Institute, Mount Sinai HospitalTorontoCanada; ^2^ Dalla Lana School of Public HealthUniversity of TorontoTorontoCanada; ^3^ Hospital for Sick Children Research InstituteTorontoCanada; ^4^ Department of Statistical SciencesUniversity of TorontoTorontoCanada

**Keywords:** bootstrap, cohort studies, DCCT/EDIC Genetics Study, genotype, phenotype, selection bias, survival analysis

## Abstract

The “winner's curse” is a subtle and difficult problem in interpretation of genetic association, in which association estimates from large‐scale gene detection studies are larger in magnitude than those from subsequent replication studies. This is practically important because use of a biased estimate from the original study will yield an underestimate of sample size requirements for replication, leaving the investigators with an underpowered study. Motivated by investigation of the genetics of type 1 diabetes complications in a longitudinal cohort of participants in the Diabetes Control and Complications Trial/Epidemiology of Diabetes Interventions and Complications (DCCT/EDIC) Genetics Study, we apply a bootstrap resampling method in analysis of time to nephropathy under a Cox proportional hazards model, examining 1,213 single‐nucleotide polymorphisms (SNPs) in 201 candidate genes custom genotyped in 1,361 white probands. Among 15 top‐ranked SNPs, bias reduction in log hazard ratio estimates ranges from 43.1% to 80.5%. In simulation studies based on the observed DCCT/EDIC genotype data, genome‐wide bootstrap estimates for false‐positive SNPs and for true‐positive SNPs with low‐to‐moderate power are closer to the true values than uncorrected naïve estimates, but tend to overcorrect SNPs with high power. This bias‐reduction technique is generally applicable for complex trait studies including quantitative, binary, and time‐to‐event traits.

## Introduction

Genetic association studies conducted by large‐scale genotyping of genetic variants such as single‐nucleotide polymorphisms (SNPs) in existing well‐characterized longitudinal cohorts are an attractive approach to investigate complex traits. In addition to examining repeated measurements of binary or quantitative traits, investigators can track phenotypic changes through time and detect the points of development of disease relevant events. Such events may include age at onset of disease, time to mortality, or time to secondary complications of disease. Notable examples include the Framingham Heart Study, the Women's Genome Health Study, and the Women's Health Initiative [Cupples et al., 2007; Prentice and Anderson, 2008; Ridker et al., 2008].

As in the analysis of disease status or a quantitative trait, the selection of a genetic variant associated with a time‐to‐event trait according to a large test statistic introduces optimistic bias into the association parameter estimate. This exaggeration has previously been referred to as the Beavis effect [Sun and Bull, 2005; Xu, 2003] or the winner's curse bias [Kraft, 2008; Voight and Cox, 2004] and is detrimental to the interpretation of detected associations and to the design of replication studies. It is a consequence of using the same sample for both gene discovery and effect size estimation [Göring et al., 2001]. This is precisely where the problem arises—typically, investigators are interested in the magnitude of the genetic association parameter only after an SNP has passed some statistical selection threshold or is ranked among the top SNPs. On average, this practice of threshold and/or ranking selection produces exaggerated association estimates [Faye et al., 2011]. Estimates of sample size required to detect a statistically significant association is a function of the postulated effect size. If the effect size estimate used in the design stage of a replication study is exaggerated, then the study will be underpowered to detect the true association, if one in fact exists, and reproducibility will be compromised.

In the context of high‐dimensional multiple testing, adjustment of association estimates for winner's curse inflation is critical for interpretation and replication of findings, including large‐scale candidate gene studies, genome‐wide association studies (GWAS), and next‐generation sequencing whole‐genome analyses. Although ranking and threshold selection bias do not apply if independent samples are available for gene discovery vs. parameter estimation, splitting study participants into two independent groups is generally inefficient. Sun and Bull [2005] proposed a nonparametric bootstrap resampling method that estimates selection bias by mimicking a design with independent detection and estimation samples within each bootstrap replicate. Wu et al. [2006] and Yu et al. [2007] applied the bootstrap approach in genetic linkage analysis and two‐stage study design, respectively. The method effectively adjusts simultaneously for threshold selection arising from use of stringent significance criteria and ranking selection arising from maximization of the association statistics over the genome or a gene set, as demonstrated in extensions for disease status and quantitative traits developed in the genome‐wide (GW) association setting [Faye et al., 2011; Sun et al., 2011].

Likelihood‐based approaches, also applied without the requirement of an independent sample, account for threshold selection by conditioning on the probability that the test statistic for a single SNP exceeds a critical value threshold. Available conditional likelihood methods for bias‐reduced estimation were developed mainly for case‐control designs, and do not address ranking bias because they deal with a single SNP at a time [Ghosh et al., 2008; Xiao and Boehnke, 2009, 2011; Zhong and Prentice, 2008, 2010; Zollner and Pritchard, 2007]. These methods are designed to reduce bias in estimates of genetic association for binary or quantitative traits, but comparable methods for survival models have not been investigated. The approximate conditional likelihood procedure of Ghosh et al. [2008] however, can be applied to a broader class of phenotypes because it requires only summary statistics from standard statistical procedures. Faye et al. [2011] also investigated a single‐SNP (SS) bootstrap method that performs similarly to the SS likelihood methods in GWAS, concluding that SS methods perform well only when power to detect association is high.

In this report, we extend the bootstrap bias‐reduction methods to the analysis of time‐to‐event traits under the Cox proportional hazards (PH) model and apply the methods in a large‐scale candidate gene association analysis of complications in type 1 diabetes (T1D) [Al‐Ketab et al., 2008]. Although extensive prior evaluations of bias‐reduction methods for binary traits in the GWAS setting would predict similar performance for time‐to‐event traits, power to detect SNPs associated with time to event depends on the number of events, which may be more modest than in a case‐control design. Moreover, the ratio of null to true‐positive SNPs in candidate gene studies can be different from that in GWAS, particularly in large‐scale studies with well‐chosen candidates, which is likely to alter the performance properties of the bootstrap method [Wu et al., 2006]. In simulation studies based on the application dataset, we therefore evaluate the performance of the bias‐reduction methods across a range of SNP associations that differ in underlying power.

## Methods

### Power and Bias in Genetic Association with Time to Event

The magnitude of the winner's curse bias is a function of the underlying power to detect an association. This form of selection bias is especially prevalent in studies with low power, in which a genetic variant selected as statistically significant or being top ranked tends to have a test statistic that is extreme with respect to the true underlying distribution. As detailed in the Supporting Information, we derive a simple approximation for the expected bias based on expressions from Therneau and Grambsch [2000, Section 3.6] for power of a test of association under a Cox PH survival model. This shows that the magnitude of bias in the estimated log hazard ratio (logHR) depends largely on power. Bias increases with more stringent significance threshold and lower minor allele frequency (MAF) of the SNP, whereas bias decreases with larger magnitude of the true genetic association (logHR) and larger number of events (supplementary Fig. S1). Supplementary Figure S1 also illustrates that truly associated and null SNPs can produce similar effect estimates.

### GW Bootstrap Bias‐Reduction Procedure

As implemented for genetic association analysis [Sun et al., 2011], the general nonparametric bootstrap bias‐reduction method accounts for MAF variation among SNPs and for negative correlation between each bootstrap sample and its complement. Following Faye et al. [2011], we let ${\hat{\beta }}_{N(k)}$ (*k* = 1, …, *K*, where *K ≥*1) represent the logHR estimate of the *k*th‐ranked SNP from Cox PH model analysis of the original sample of size *n*, which we term the naïve estimate; *p*(*k*) is the MAF of the *k*th‐ranked SNP. Assuming these *K* SNPs are selected by significance threshold and/or ranking criteria, the naïve estimate will tend to be exaggerated because the same sample was used for detection/ranking and for estimation of the associations.

For ease of implementation, if any of the naïve estimates ${\hat{\beta }}_{N(k)}$ are negative, the bootstrap method begins by recoding the SNP genotype to ensure that all of the association estimates are in the positive direction. A series of bootstrap sample replicates, indexed by *i* = 1, …, *B*, are obtained by resampling from the original study observations. For replicate *i*, the within‐sample estimate includes *n* observations sampled with replacement into bootstrap sample *i*, while the out‐of‐sample estimate includes the remaining observations not sampled into bootstrap sample *i*. The same significance threshold and/or ranking criteria are applied, but the SNPs selected in a bootstrap sample need not be the same as those selected in the original sample. We let ${\hat{\beta }}_{Di(k)}$ be the within‐sample logHR estimate of the *k*th‐ranked SNP in bootstrap replicate *i* with variance estimate ${{\hat{\sigma }}^{2}}_{Di(k)}$ and ${\hat{\beta }}_{Ei(k)}$ be the out‐of‐sample logHR estimate of the same SNP with variance estimate ${{\hat{\sigma }}^{2}}_{Ei(k)}$. *p_i_*_(_ *_k_*_)_ is the MAF of the *k*th‐ranked SNP in bootstrap replicate *i*; the MAF for this SNP is estimated in the original data.

The bias‐reduced parameter estimate of Faye et al. [2011], adjusted for negative correlation between ${\hat{\beta }}_{Di(k)}$ and ${\hat{\beta }}_{Ei(k)}$ as well as for SNP MAF, is given by

(1) $$\begin{array}{c} {\hat{\beta }}_{boot(k)}^{*}={\hat{\beta }}_{N(k)}-\frac{\frac{1}{B_{\left(k\right)}}\sum_{i=1}^{B_{\left(k\right)}}\;\left({\hat{\beta }}_{Di(k)}-{\hat{\beta }}_{Ei(k)}^{*}\right)\sqrt{2p_{i(k)}\left(1-p_{i(k)}\right)}}{\sqrt{2p_{\left(k\right)}\left(1-p_{\left(k\right)}\right)}} \end{array}$$

(2) $${\hat{\beta }}_{Ei(k)}^{*}={\hat{\beta }}_{Ei(k)}-\frac{{\hat{\sigma }}_{DEi(k)}}{{{\hat{\sigma }}^{2}}_{DEi(k)}}\left({\hat{\beta }}_{Di(k)}-{\hat{\beta }}_{Ni(k)}\right)$$

The bias‐reduction procedure averages over $B_{\left(k\right)}\left(\leq B\right)\;$ bootstrap samples that have at least *k* SNPs passing the same selection criterion as the original sample. In deriving Equation (1), we take an average of the differences between ${\hat{\beta }}_{Di(k)}$ and ${\hat{\beta }}_{Ei(k)}$ as an estimate of the magnitude of the threshold/ranking selection bias for the *k*th‐selected SNP in the original sample, treating the within‐sample and the out‐of‐sample observations as independent samples. That is, in each bootstrap replicate, the within‐sample observations imitate a sample used for gene detection, while the out‐of‐sample observations imitate a separate sample used for association parameter estimation.

The within‐sample and the out‐of‐sample estimates, however, are somewhat negatively correlated because the total sample is fixed. Faye et al. [2011] derived a correction for the resulting negative correlation between ${\hat{\beta }}_{Di(k)}$ and ${\hat{\beta }}_{Ei(k)}$ that depends on the estimated variances ${{\hat{\sigma }}^{2}}_{Di(k)}$ and ${{\hat{\sigma }}^{2}}_{Ei(k)}$, and the estimated covariance ${\hat{\sigma }}_{DEi(k)}$ as shown in Equation (2), where ${\hat{\beta }}_{Ni(k)}$ is the original naïve sample estimate for the *k*th‐ranked SNP detected in the *i*th bootstrap sample. Note that ${\hat{\beta }}_{Ni(k)}$ differs from ${\hat{\beta }}_{N(k)}$, the estimate for the *k*th‐ranked SNP in the original sample.

The bias in the logHR estimate induced by selection is inversely proportional to the square root of the genotypic variance of the associated SNP (i.e., variance = 2*p*(1 − *p*), where *p* = MAF, see Supporting Information for details), with higher sampling bias for lower frequency SNPs. In Equation (1), each term in the bootstrap sample average is rescaled using the MAF of the *k*th‐ranked SNP in the bootstrap sample *i* (*p_i_*_(_ *_k_*_)_) [Faye et al., 2011; Sun et al., 2011]. It is important to account for allele differences across SNPs by both *p_i_*_(_ *_k_*_)_ and *p*_(_ *_k_*_)_ *_,_* (the MAF of the *k*th‐ranked SNP in the original sample) because the *k*th‐ranked SNP in the bootstrap sample may differ from the *k*th‐ranked SNP in the original sample. The shrinkage estimator ${{\hat{\beta }}^{*}}_{boot(k)}$ defined in Equation (1), which reduces the magnitude of the naïve estimate by the bootstrap estimate of bias, is truncated at the null to avoid a change in the direction of association in the bias‐reduced estimate as compared to the naïve estimate.

#### Implementation

We implemented time‐to‐event bootstrap bias‐reduction using the open‐source software: BR‐squared (Bootstrap Resampling Bias Reduction; http://www.utstat.toronto.edu/sun/Software/BR2/) [Sun et al., 2011] and the open‐source PLINK software (http://pngu.mgh.harvard.edu/∼purcell/plink/) [Purcell et al., 2007]. Both BR‐squared and PLINK are efficient software packages, designed to handle large datasets with a large number of SNPs (see Sun et al. [2011] for details). Analysis of quantitative or binary traits by linear or logistic regression, respectively, is built into the BR‐squared software. To apply the Cox PH model, BR‐squared can make use of the R “survival” function [Therneau, 2011] as an R plug‐in. Example code with the R plug‐in is provided in the Supporting Information.

## Application to the Diabetes Control and Complications Trial/Epidemiology of Diabetes Interventions and Complications (DCCT/EDIC) Genetics Study

Our extension of bias‐reduction methods to the analysis of time to event is motivated by the DCCT/EDIC Genetics Study which was designed to investigate the association of SNPs in a large set of candidate genes with time to complications of T1D in DCCT participants [Al‐Ketab et al., 2008]. The DCCT/EDIC study is a long‐term follow‐up study of randomized trial participants [DCCT Research Group, 1993; EDIC, 1999] in which two cohorts of individuals with T1D (primary prevention and secondary intervention) were randomly assigned to receive conventional or intensive therapy. As detailed in the methods of Al‐Kateb et al. [2008], tagging SNPs within 5 kb flanking either side of each candidate gene were selected so as not to be in strong linkage disequilibrium, and a total of 1,441 SNPs were genotyped by a custom Illumina GoldenGate Beadarray assay in 1,361 white probands. (Absence of admixture was confirmed subsequently using GWAS principal components.) These authors reported analysis of 1,213 SNPs with MAF ≥ 5% in 201 candidate genes, using a Cox PH model for incidence of severe nephropathy, including known risk factors and study design factors as explanatory covariates. An association was detected with rs17880135 in the *3*′ region of superoxide dismutase 1 (*SOD1*) gene (hazard ratio [HR] = 2.62, *P* = 5.6 × 10^−5^). However, as the authors noted, this HR estimate is expected to be optimistic because a large number of SNP associations were examined.

**Figure 1:**
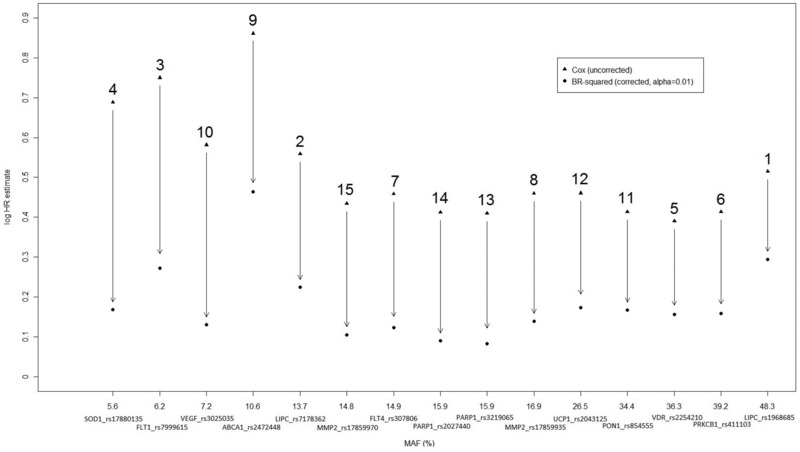
Log HR estimates under a Cox proportional hazards model analysis of time to severe nephropathy for the top 15 SNPs in the DCCT/EDIC Genetics Study dataset, including 1,361 individuals. The horizontal axis corresponds to the minor allele frequency (MAF), with each SNP annotated with gene name and rs number. The number above each vertical arrow indicates the SNP ranking according to the *P*‐value of the original test of association (reported in Table 1). The vertical arrows quantify the reduction in the logHR by the genome‐wide bootstrap method: the percentage reduction varies with MAF from 43.1 (at MAF = 48.3%) to 80.5% (at MAF = 15.9%).

We analyzed 1,361 individuals for association with time to diabetes complication including 115 diagnosed with severe nephropathy during follow‐up. Our univariable Cox PH model analysis, including only one SNP at a time and no other covariates, detected 15 SNPs (MAF ≥ 5%) statistically significant at the 1% level. We applied the BR‐squared time‐to‐event implementation to estimate bias‐reduced logHR ratios for these SNPs (Fig. 1). The differences between naïve and GW bootstrap bias‐reduced estimates of the logHRs for the top 15 SNPs (Table 1) exhibit considerable variation in bias reduction across SNPs with percentage of reduction in the logHR estimates ranging from 43.1% to 80.5%. For example, rs3025035 in *VEGFA* with an MAF of 7.2% has an uncorrected naïve logHR of 0.58 (*P* = 0.0053) compared to a bias‐reduced value of only 0.13, corresponding to a 77.6% reduction. In contrast, bias reduction for rs2472448 in *ABCA1* (MAF = 10.6%) with a large naïve logHR estimate is more modest (46.3%). *Post hoc* power calculations in the last two columns of Table 1 indicate that power and sample size estimates for replication can differ markedly.

**Table 1:** DCCT/EDIC study naïve and genome‐wide bootstrap bias‐reduced logHR estimates (taken in absolute value)

				Univariable models					
Gene		SNP	MAF (%)	*P*‐value	Naïve logHR estimate	Genome‐wide bootstrap estimate	Percentage reduction of naïve by genome‐wide bootstrap	Power by naïve (%)	Power by genome‐wide bootstrap (%)
1	*LIPC*	rs1968685	48.3	2.22 × 10^−4^	0.51	0.29	43.1	90.1	35.3
2	*LIPC* a	rs7178362	13.7	2.36 × 10^−4^	0.56	0.22	60.1	63.4	7.7
3	*FLT1*	rs7999615	6.2	6.13 × 10^−4^	0.75	0.27	64.0	57.3	5.7
4	*SOD1* a	rs17880135	5.6	8.18 × 10^−4^	0.69	0.17	75.4	43.2	2.4
5	*VDR*	rs2254210	36.3	2.61 × 10^−3^	0.39	0.16	59.0	60.4	7.9
6	*PRKCB1*	rs411103	39.2	3.82 × 10^−3^	0.41	0.16	61.0	67.7	8.2
7	*FLT4*	rs307806	14.9	3.85 × 10^−3^	0.46	0.12	73.9	46.4	3.8
8	*MMP2*	rs17859935	16.9	4.36 × 10^−3^	0.46	0.14	69.6	53.0	3.8
9	*ABCA1* a	rs2472448	10.6	5.00 × 10^−3^	0.86	0.46	46.5	91.5	32.2
10	*VEGFA*	rs3025035	7.2	5.34 × 10^−3^	0.58	0.13	77.6	35.4	1.9
11	*PON1* a	rs854555	34.4	6.22 × 10^−3^	0.41	0.17	58.5	64.7	8.8
12	*UCP1*	rs2043125	26.5	6.52 × 10^−3^	0.46	0.17	63.0	69.3	7.5
13	*PARP1* a	rs3219065	15.9	7.75 × 10^−3^	0.41	0.09	78.0	38.0	1.9
14	*PARP1* a	rs2027440	15.9	8.18 × 10^−3^	0.41	0.08	80.5	38.0	1.6
15	*MMP2*	rs17859970	14.8	9.99 × 10^−3^	0.43	0.10	76.7	40.8	2.1

a Minor allele is the risk allele, otherwise major allele is associated with risk.

Analysis of time to severe nephropathy (1,361 individuals with 115 events) based on a significance threshold selection criterion of *P* < 0.01. SNPs with MAF ≤ 5% are excluded. Bias reduction ranges from 43.1% to 80.5%. The final two columns display *post hoc* power calculations for a similar sample assuming logHRs are set to the value of the naïve or the genome‐wide bias‐reduced estimates.

Individual‐level risk scores constructed as a linear combination of risk allele counts weighted by SNP effect estimates are also subject to a winner's curse bias. In the Cox PH model, the linear predictor in the exponential term of the hazard model corresponds to a risk score, and the difference in risk scores between individuals corresponds to a logHR. We calculated naïve and GW bootstrap risk scores for each of the 1,361 DCCT/EDIC individuals. A higher individual score suggests a higher risk of severe nephropathy. Here use of bootstrap resampling is roughly analogous to use of cross‐validation to reduce over‐fitting bias due to variable selection. As expected, the GW risk score exhibits shrinkage relative to the naïve risk score (supplementary Fig. S2a). The maximum risk score is reduced from 12.01 to 4.20, and from 4.40 to 1.37 for the minimum; the HR comparing the highest to the lowest risk individual is reduced from a naïve value of 2,018.3 to a GW bootstrap value of 16.9. The ranking of individuals according to their risk score can be altered by the GW bias reduction, especially for individuals in the central part of the risk score distribution (supplementary Fig. S2b).

## Simulation Studies

### Bias‐Reduced Association Estimators

To evaluate the GW bootstrap estimator in comparison to the uncorrected naïve estimator in a large‐scale candidate gene study of time to event, we conducted simulation studies under a Cox PH model based on the DCCT/EDIC genetic data. We also compare the GW bootstrap estimator to two computationally attractive methods that address threshold but not ranking bias: a SS bootstrap estimator and a conditional likelihood estimator (see Supporting Information for details). The GW bootstrap, as defined in Equation (1) above, averages across possibly different SNPs selected in each of the bootstrap samples by genome‐wide association (or candidate‐gene) analysis. The SS bootstrap similarly averages across repeated bootstrap sample replicates, but for each SNP the bias estimate is based on the difference between the detection and the estimation logHR values for that same SNP, averaging only the replicates where the SNP association test meets the significance threshold. It effectively proceeds as if there is only that one SNP being tested for association. The conditional likelihood estimator recommended by Ghosh et al. [2008] is the so‐called compromise estimator for bias reduction, the average of the simple conditional maximum likelihood estimate and the mean of the normalized conditional likelihood estimate. Here as well, the method does not account for ranking selection, considering only one SNP at a time, and conditioning is on the significance of that SNP alone.

### Design

In the simulations, we used the observed DCCT/EDIC study genotypes to generate time to event, with time from trial entry to diagnosis of nephropathy as the outcome of interest. To cover a wider spectrum of power to detect a true SNP association across the SNPs included in the generating model for simulated datasets, we used a sampling approach to expand the sample size from the 1,361 individuals in the original DCCT/EDIC study to 5,444 individuals. An additional 4,053 individuals were obtained such that the genotypes of each “new” individual were a combination of the genotypes of two individuals randomly sampled with replacement from the original 1,361 individuals. To maintain LD structure, 729 of the SNPs were taken from the first sampled individual and 735 different SNPs were taken from the second, such that SNPs within any given candidate gene region were from a single individual. In this way, MAF was maintained within half a percentage point of the values in the original data. The observed covariate values for the second sampled individual were retained as the covariate values for a “new” individual.

The SNPs specified to have true‐positive associations in the data generating model included 15 common SNPs (MAF ≥ 5%) and four low‐frequency SNPs (2% < MAF < 5%). The former corresponded to the top SNPs in the DCCT/EDIC data analysis (Table 1), and the latter were included to represent the presence of undetectable true‐positive associations contributing to polygenetic association. In simulation Studies 1 and 2, the logHR parameter for each SNP was set to be the absolute value of the GW bootstrap estimate obtained in univariable analysis of the original data (based on 100 bootstrap samples, a *P*‐value threshold of 0.01, and MAF > 2%). Parameter values for three covariates: “sex” (logHR = −0.59), “treatment” (logHR = −1.48), and “cohort” (logHR = 0.73) were set to their multivariable logHR estimates from the original DCCT/EDIC dataset with stratification by year of entry. In simulation Study 3, the data generating model was altered by setting the parameter values to have the same sign as the original estimates, which yielded a different combination of risk alleles.

For each individual in a simulated dataset, we generated a time to event from their covariate and genotype data using an SAS implementation of the method described in Bender et al. [2005]. An individual‐specific risk score was calculated from the linear combination of his/her covariates and genotypes with corresponding parameter values. We included strata for year of trial entry with covariates for sex, treatment group, and cohort, as well as for the 19 SNP genotypes, coded additively. The baseline cumulative hazard was estimated for each stratum using observed time to event for 1,361 individuals from the original EDIC/DCCT dataset. The event‐time variable for each individual was computed using an equation equivalent to Bender's Equation (6) [2005], and compared to a standard uniform variate. Where the uniform variate fell below the last step of the estimated survival curve, the event time was set to censored at the last visit. This generated a censoring pattern similar to that observed in the original dataset. In the DCCT/EDIC study, very few individuals dropped out during follow‐up, so we did not simulate any mid‐study dropouts.

In each simulated dataset of 5,444 individuals thus generated, each of 1,213 SNPs with MAF ≥ 5% was analyzed separately in a univariable model with additive genotype coding, regardless of whether the SNP was a true positive according to the generating model or a null association. Because time to nephropathy was generated under a multivariable model, but the SNP genotypes were analyzed one at a time under a misspecified univariable model, the underlying marginal regression parameter value for each true‐positive SNP was determined as the average of the univariable fitted logHR value over all replicates. This included datasets in which the SNP met statistical significance criteria as well as those in which it did not. Empirical power for each true‐positive SNP was estimated as the frequency of detection by the large sample Wald test (*P* < significance threshold) divided by the total number of replicated datasets. For each of the significant SNPs detected in a dataset, a bias‐reduced logHR was estimated. In total, 5,000 simulated datasets were generated as replicates to investigate the effect estimate distributions for true‐positive and false‐positive SNPs. Simulation Studies 1 and 2 differed only in the significance levels used to select SNPs, *P* < 5 × 10^−5^ and *P* < 0.01, respectively. We specified the stricter significance threshold in Studies 1 and 3 to account for multiple testing, and relaxed the threshold in Study 2 to assess sensitivity of results to the threshold specification.

The empirical mean across replicates was calculated for the naïve and bias‐reduced logHR estimates for the true‐positive SNPs with MAF ≥ 5%. We also examined the distribution of logHRs for each of the true‐positive SNPs, and for the false‐positive SNPs grouped into categories according to MAF (≥5%). To obtain a purer assessment of false‐positive associations, 254 SNPs in linkage disequilibrium (*r* ^2^ > 0.2) with those in the generating model were excluded from the false‐positive distributions.

### Results

#### GW Method

As evident in summary statistics for the true positive common SNPs reported in Table 2 for simulation Study 1 (where row entries are ordered by empirical power under the significance threshold of *P* < 5 × 10^−5^), power is higher for SNPs with larger mean fitted marginal logHR and higher MAF. As empirical power falls below 65%, the naïve logHR values increasingly overestimate the marginal fitted value on average, while the GW bootstrap estimates are closer to the true effect size. For instance, among the 463 statistically significant realizations for the SNP with MAF of 5.8%, which is detected with power of only 9.3%, the mean naïve logHR estimate is 0.34, much larger than the marginal fitted logHR of 0.21 (supplementary Table S1). The mean GW bootstrap estimate for this SNP is 0.23, corresponding to a 34.0% reduction in the naïve estimate, an under‐correction with a modest absolute bias of 0.02.

**Table 2:** Simulation Study 1 summary statistics for the genome‐wide bootstrap, conditional likelihood, and single‐SNP bootstrap estimates (significance threshold *P* < 5 × 10^−5^)

				Mean bias in logHR estimates				
SNP MAF	Data‐generated logHR	Empirical power (%)	Mean fitted logHR	Uncorrected naïve	Genome‐wide bootstrap	Conditional likelihood	Single‐SNP bootstrap	No. of datasets selected
10.3	0.50	99.5	0.50	0.00	−0.07	0.00	0.00	4,974
34.9	0.19	77.3	0.22	0.01	−0.04	0.01	0.00	3,863
26.7^a^	0.22	65.5	0.20	0.03	−0.03	0.02	0.01	3,275
17.1	0.17	55.7	0.26	0.04	−0.03	0.03	0.01	2,785
15.0	0.13	52.2	0.27	0.04	−0.03	0.03	0.02	2,611
13.7^a^	0.23	50.0	0.22	0.04	−0.03	0.03	0.02	2,499
48.8^a^	0.27	17.2	0.14	0.05	0.00	0.04	0.03	861
39.0^a^	0.21	10.0	0.12	0.07	0.01	0.06	0.04	500
5.8^a^	0.19	9.3	0.21	0.13	0.02	0.12	0.09	463
15.8^a^	0.09	8.9	0.15	0.09	0.01	0.08	0.06	447
15.8^a^	0.13	8.4	0.15	0.09	0.01	0.08	0.06	418
14.6	0.14	0.7	0.12	0.16	0.08	0.15	0.13	36
36.3	0.14	0.6	0.07	0.12	0.07	0.11	0.10	29
6.8	0.27	0.2	0.16	0.26	0.15	0.23	0.19	11
6.9	0.12	0	0.09	NA	NA	NA	NA	0

a Minor allele is the risk allele, otherwise major allele is associated with risk.

Comparison with the *naïve* Cox PH estimates for 15 SNPs generated to have association with time to severe nephropathy in a sample of 5,444 individuals. The rows are ordered by empirical power, which is the proportion of simulated datasets in which the SNP was detected as significant out of 5,000 replications. Mean bias is calculated as the difference between the mean fitted logHR in all datasets and the mean logHR in selected datasets.

**Figure 2:**
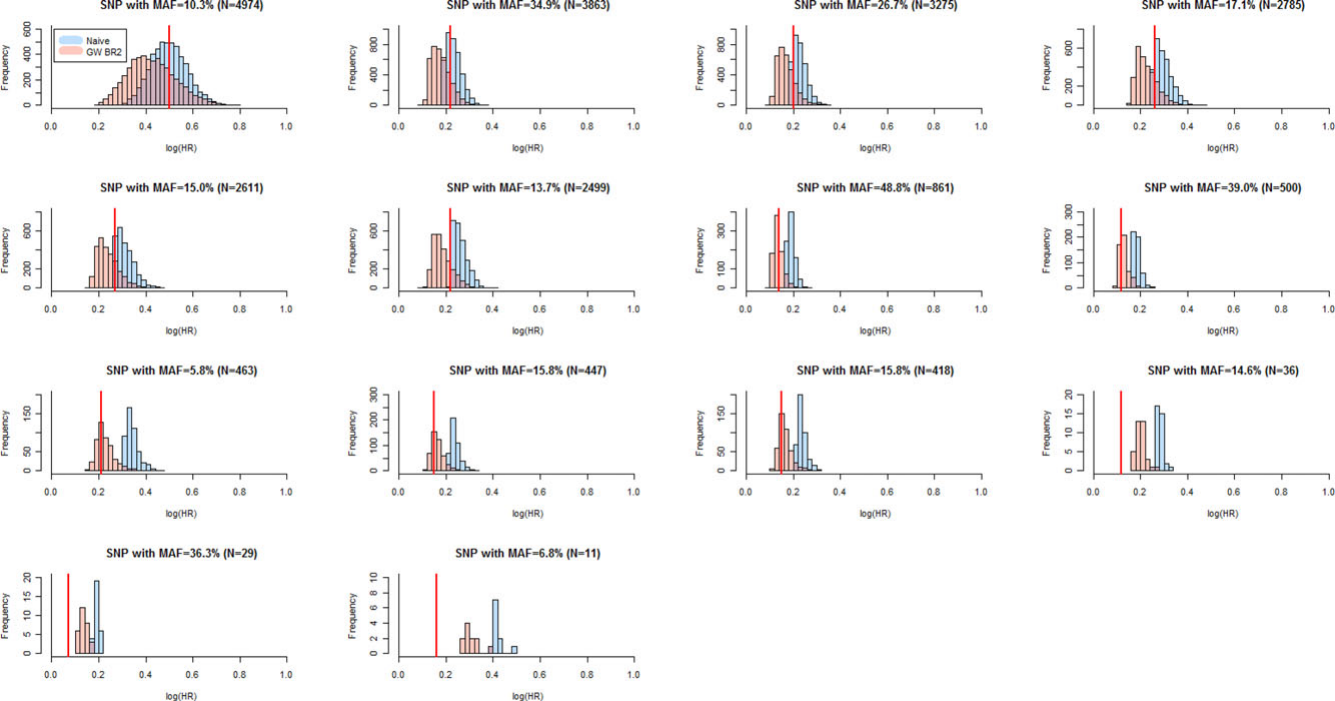
Simulation Study 1 genome‐wide bootstrap estimates for true‐positive SNPs (significance threshold *P* < 5 × 10^−5^). Comparison of distributions of *genome‐wide bootstrap* (transparent red GW BR2) and uncorrected *naive* (transparent blue) logHR estimates of true‐positive SNPs with MAF ≥ 5% out of the 5,000 replications of a sample of 5,444 subjects. The vertical, solid red line denotes the fitted logHR averaged across unselected datasets. The SNPs are ordered by number of simulation datasets (*N*) in which the SNP was detected as statistically significant (see Table 2).

**Figure 3:**
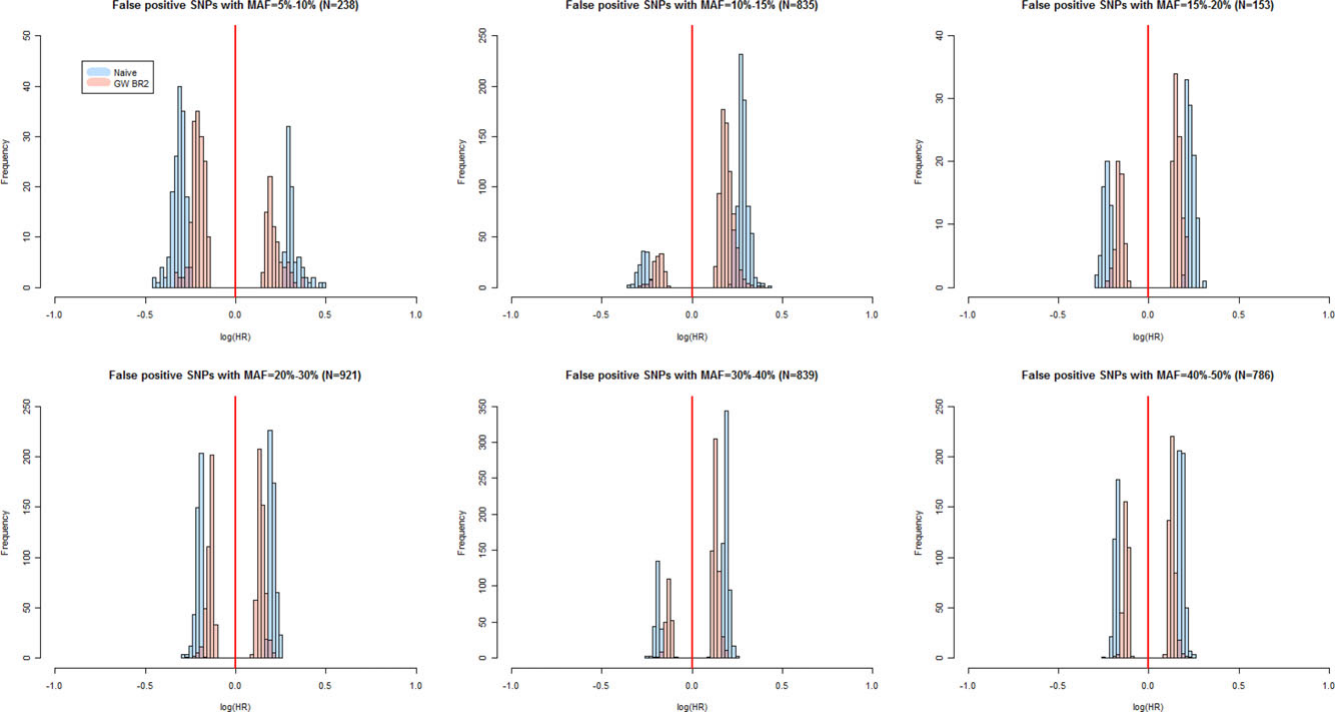
Simulation Study 1 genome‐wide bootstrap estimates for false‐positive SNPs (significance threshold *P* < 5 × 10^−5^). Comparison of distributions of *genome‐wide bootstrap* (transparent red GW BR2) and uncorrected *naive* (transparent blue) logHR estimates of false‐positive SNPs in a sample of 5,444 subjects, stratified by MAF categories. False‐positive SNPs are those found to be statistically significant among 5,000 replications and not in the same gene as any of the SNPs in the model used for data generation. The vertical solid red line denotes the null reference value.

The distributions of estimates for the 14 true‐positive SNPs with nonzero empirical power (*P* < 5 × 10^−5^) demonstrate that power is a critical factor (Fig. 2). Among SNPs with power below 65%, the distribution of the naïve estimates exceeds the marginal fitted value, while the distribution of the GW bootstrap estimates usually includes it. At power greater than 70%, the naïve estimates are nearly unbiased, while the GW bootstrap estimates tend to become conservative on average, with about 25% of the estimates larger than the true effect size. As an extreme example, consider the SNP in the first row of Table 2 where the underlying effect size is quite large and the empirical power (99%) at a level rarely achieved in practice. Averaged across 4,974 statistically significant associations (*P* < 5 × 10^−5^), the mean naïve logHR estimate of 0.50 (HR = 1.65) is the same as the underlying marginal fitted effect size, while the mean GW bootstrap value is 0.43 (HR = 1.54). On the other hand, across all power levels, the GW bootstrap estimates are less likely to have values larger than the marginal effect size (Fig. 2). At lower MAF, the bias in the naïve estimates can be quite large, and moreover, in each MAF category of false‐positive SNPs, the distribution of the GW bootstrap estimates is much closer to the null than that of the naïve estimates (Fig. 3).

When the significance threshold is less stringent (*P* < 0.01), the power to detect association is higher for the true‐positive SNPs and the naïve estimates are less biased on average (Study 2: supplementary Table S4, supplementary Fig. S9), but this comes at the cost of more false‐positive detections (supplementary Fig. S10). Furthermore, for SNPs with less than 55% power, the GW bootstrap method has smaller absolute mean bias than the naïve estimates. For Study 3 where true‐positive parameter effects include a different combination of minor and major allele associations, results are similar generally to those of Study 1 (supplementary Table S5, supplementary Fig. S11). Here again, the GW bootstrap method is effective for SNPs with power less than 65%. False‐positive distributions are also similar to the patterns observed in Figure 3, with decreasing variance as MAF increases (supplementary Fig. S12).

#### SS Methods

The conditional likelihood estimator works directly with the Cox PH regression results, separately for each of the SNPs, so it is easy to compute. For true‐positive SNPs with power greater than 50%, we observed substantial overlap of the distribution of the conditional likelihood estimates with those of the naïve and the GW bootstrap estimates, although the variance of the conditional estimates is wide with a long upper tail indistinguishable from that of the naïve estimates (supplementary Figs. S3 and S5). This method tends to be unsatisfactory for bias reduction of false‐positive associations (supplementary Figs. S4 and S6) and true‐positive SNPs with power less than 50% (Table 2). The SS bootstrap estimates are less biased than the conditional likelihood estimates (Table 2). For SNPs with power greater than 50%, the SS bootstrap yields estimates closer on average to the true underlying genetic effect than the GW bootstrap, but the SS bootstrap estimates have increasingly positive bias as power falls below 50% and the majority of the SS estimates are larger than the marginal fitted effect size (Table 2, supplementary Figs. S7 and S8).

**Figure 4:**
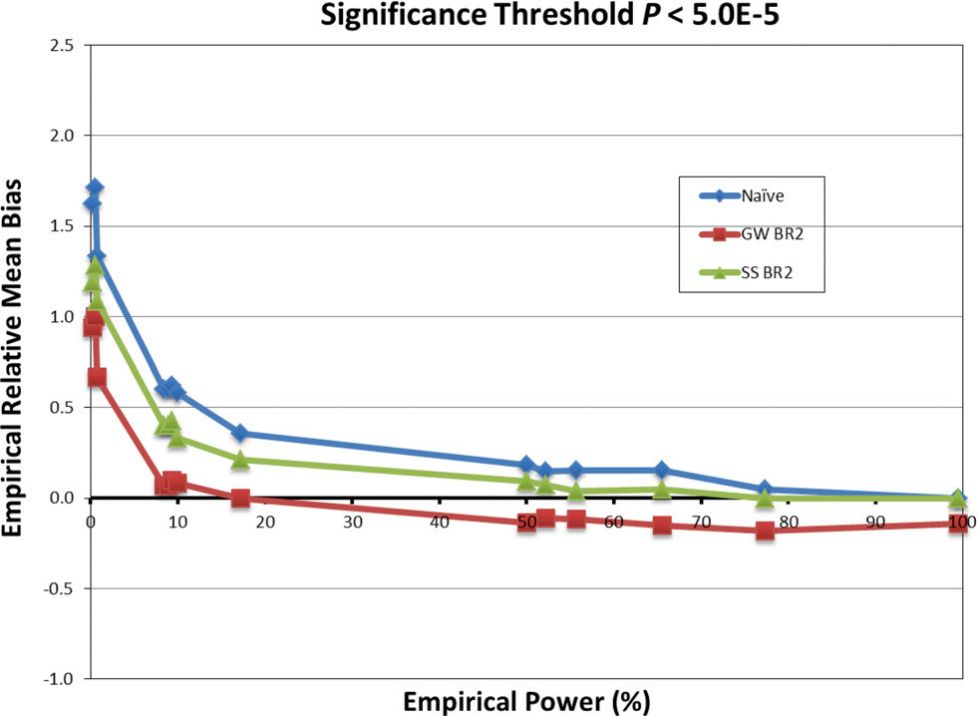
Simulation Study 1 relative mean bias comparison of the *genome‐wide* (GW BR2) and *single‐SNP* (SS BR2) bootstrap estimates with the *naïve* estimates for 14 SNPs with MAF ≥ 5% generated to have association with time to severe nephropathy in a sample of 5,444 subjects. Relative mean bias is calculated as Mean bias/mean fitted effect size, where means are taken over 5,000 replications (values derived from supplementary Tables S1 and S3).

When power is high to detect a true‐positive SNP, the SS bootstrap estimates are closer to the marginal fitted mean on average, but are optimistic for low power true positives and false positives (Fig. 4). Consideration of the SS bias‐reduction term illustrates why the SS bootstrap does not sufficiently correct a low power or false‐positive SNP. In the SS procedure, only the SNP of interest is tested in each of the bootstrap samples. Assuming it is detected as significant in *B* of the bootstraps, the effect size bias estimate (ignoring the correlation correction) is

$$\Delta_{SS}=\frac{1}{B}\sum_{i=1}^{B}\left({\hat{\beta }}_{Di}-{\hat{\beta }}_{Ei}\right)$$

For a false‐positive SNP detected in the original sample when threshold bias is high, both effect estimates, within‐sample and out‐of‐sample, will be exaggerated. Then the bootstrap sample difference, ${\hat{\beta }}_{Di}-{\hat{\beta }}_{Ei}$, will tend to underestimate the bias, and Δ*_SS_* will be too small.

#### Risk Score Estimation

Based on generating models and the mean of the naïve and GW bootstrap estimates obtained in simulation Studies 1–3 (supplementary Tables S1, S4, and S5), we calculated the corresponding expected risk scores according to the linear predictor equation: $S=\sum_{k=1}^{m}\beta_{k}\times \;q_{k}$ with $\beta_{k}$, the logHR for the risk allele of SNP *k*, as specified in the generating model or as determined by the mean naïve or GW bootstrap estimate from the simulations. Here, $q_{k}$ is the expected value of an additively coded genotype for the risk allele, and under Hardy‐Weinberg equilibrium, $q_{k}=2p_{k}$, where $p_{k}$ is the risk allele frequency. Overall, the naïve expected risk score overestimates the risk score under the generating model, and the GW bootstrap expected risk score is closer to the generating risk score, but can over or underestimate depending on the *P*‐value threshold, and the risk allele frequency in the generating model (Table 3).

**Table 3:** Expected risk scores based on the naïve and genome‐wide (GW) bootstrap mean logHR estimates from 5,000 simulation datasets compared to the risk score based on the generating values

		Expected risk score			
		
Simulation study	Bootstrap *P*‐value threshold	Generating	Naïve	GW	Naïve – GW difference
1	5 × 10^−5^	3.49	4.44	3.40	1.04
2	0.01	3.49	4.18	2.83	1.35
3	5 × 10^−5^	2.89	4.38	3.45	0.93

Risk scores are calculated from 15 SNPs generated to have association with risk of severe nephropathy in a sample of 5,444 individuals. Expected scores are taken over a hypothetical population with the same marginal allele frequencies as the sample of 5,444.

## Discussion

BR‐squared computation time scales linearly with the number of individuals and the number of SNPs [Sun et al., 2011]. BR‐squared is implemented to utilize a single computer with multiple CPUs or a heterogeneous computer cluster. In the DCCT/EDIC application, the CPU time for GW bias‐reduction using the R plug‐in with the BR‐squared software for a sample of 1,361 individuals, 1,213 SNPs, and *B_(k)_* = 100 bootstrap samples at the 1% significance level, was 9 min on a quad‐core 2.33 GHz processor. The method can be equally well applied in single SNP regression models with covariates, for example, multivariable models that include nongenetic covariates. At present, use of an R Cox PH survival model plugin called from the BR‐squared software is relatively slow compared to the built‐in logistic and linear regression analysis. For example, we previously reported total CPU time requirements of less than an hour for point estimation in a typical GWAS of 2,000 individuals and 1 million SNPs [Sun et al., 2011]. Computation time could be substantially improved by building a survival analysis function directly into the software. Moreover, to reduce computation in GWAS with little compromise in estimation accuracy [Sun et al., 2011], the top command in the BR‐squared software provides an option to include only the top ranked 5–10% of the original set of GWAS SNPs in the bootstrap averages (e.g., 25–50 thousand SNPs in a GWAS of 500,000 SNPs). The current BR‐squared implementation requires data formats with a single record per individual which precludes the use of methods for the analysis of survival data with time‐dependent covariates, repeated measures longitudinal data, or family data which typically require multiple‐record file formats; we plan to implement this extension in future versions of BR‐squared.

Our findings for time‐to‐event traits in the candidate gene setting are consistent with those previously reported for bootstrap bias reduction in logistic and linear regression models applied to disease status and quantitative traits in GWAS [Faye et al., 2011; Sun et al., 2011]. The GW bootstrap logHR estimates are closer to the truth than the uncorrected naïve estimates for both low and moderate power true‐positive and false‐positive SNPs, but tend to be attenuated for high power true‐positive SNPs (Fig. 4). The SS methods perform adequately for high power true‐positive SNPs, but fail otherwise because they do not account for GW SNP selection and ranking competition among all SNPs. Our GW candidate gene simulation setup specified 19 true‐positive SNPs in 14 genes, a modest proportion of the 1,441 SNPs that were successfully typed in the original study. The effect sizes were distributed such that empirical power to detect them at a significance threshold of *P* < 5 × 10^−5^ ranged from 99.5% down to 0%. In this respect, it is more similar to GWAS than a focused candidate gene study. For SNPs with power below 50%, the need for bias reduction was substantial and the GW estimates had lowest mean bias. For 50–65% power, the GW and SS estimates effectively reduced bias, with the SS estimates being optimistic and the GW estimates pessimistic. Above 65% power, the SS estimates reduced bias adequately on average without under‐correction.

As an example of the consequences for replication study sample size determination, consider the results of simulation Study 1 for the SNP with MAF = 15.0% and empirical power of 52% at *P* < 5 × 10^−5^ (row 5 of Table 2). The mean naïve, SS bootstrap, and GW bootstrap logHR estimates are 0.31, 0.29, and 0.24, respectively. Given an underlying marginal logHR of 0.27, a replication study with 630 events would have 80% power to detect an association at *P* < 0.01 (power approximation given in the Supporting Information). A replication study with 480 events designed using the naïve estimate would have 66% expected power to detect this SNP, while a study with 545 events based on the SS bootstrap estimate would have 72% power to detect this SNP but only 47% power to detect the next ranked SNP (row 6 of Table 2, empirical power of 50%). A study with 800 events based on the GW bootstrap estimate would provide more than adequate power (90%) to detect this SNP and higher ranked SNPs, and 67% power to detect the next ranked SNP.

The GW bootstrap estimator is influenced by the composition of the set of genotyped SNPs, including the number of SNPs tested, the relative proportions of null and true‐positive SNPs, and the distribution of effect sizes. In the GW bootstrap procedure, all the SNPs are tested for association with the trait in each bootstrap sample, which mimics the original GW scan. In *B_C_* of the bootstrap samples, a true‐positive SNP detected with high power in the original sample will be top ranked, but due to sampling variation, in B_C__*_ of them a null or a lower powered SNP is significant and top ranked. The GW bootstrap estimates the bias by:

$$\Delta_{GW}=\frac{B_{C}}{B}\Delta_{C}+\frac{B_{C^{*}}}{B}\Delta_{C^{*}}$$

where $\Delta_{C}=\frac{1}{B_{C}}\sum_{i\in C}\left({\hat{\beta }}_{Di}-{\hat{\beta }}_{Ei}\right)\approx \Delta_{SS}$ and $\Delta_{C^{*}}=\frac{1}{B_{C^{*}}}\sum_{i\in C^{*}}\left({\hat{\beta }}_{Di}-{\hat{\beta }}_{Ei}\right)$ (ignoring the correlation correction). The Δ_C*_ term will tend to be greater than zero because when the effect estimate of a null or lower power SNP selected in a bootstrap sample is exaggerated by selection, ${\hat{\beta }}_{Di}>{\hat{\beta }}_{Ei}$. However, unless a study is so well powered that a true‐positive SNP is detected and accurately ranked in nearly all of the bootstrap samples, the Δ*_GW_* bias estimate will tend to underestimate the effect size.

It follows that the performance of the methods can depend on the set of candidate genes chosen for the discovery study. If a well‐informed choice of candidate genes enriches the discovery study for true‐positive SNPs with good association signals, then the accuracy of the GW method is improved for a true‐positive SNP, and retained for a false‐positive SNP. Even if null SNPs are eliminated, selection bias can remain among the true‐positive SNPs and be reduced by application of the GW method. Good prior knowledge, whether based on genetic information or knowledge of biology or pathways, is helpful to the extent that it identifies a strong set of candidate genes with a smaller proportion of null associations. The accuracy of the SS method for an SNP of interest, which does not use information from any other SNPs, depends only on the magnitude of the association effect size and associated power for that SNP. Our discussion here is limited, but suggests that information about the GW distribution of effect estimates for a given trait, i.e., genetic architecture, may be informative for method choice.

For any particular SNP association detected in analysis of a single discovery dataset, it is important to keep in mind that we cannot tell whether it is a true or a false positive; when power is low, a large logHR can be observed in either case (see supplementary Fig. S1, for example). In principle, any bias‐reduction method, including the bootstrap procedures, cannot distinguish between true‐ and false‐positive detections. When the proportion of true positives is expected to be small and false positives are of concern, we recommend use of GW bootstrap estimates when multiple SNPs are being considered for replication. Providing feasibility is not in jeopardy, conservative estimates may give a better sense of the prospects for successful replication. Replication studies often aim to replicate multiple top SNPs, so sufficient power for lower ranked SNPs (smaller effect or small MAF) is critical and conservative estimates for the high‐powered SNPs is less of a concern.

In summary, statistical detection of the association of a putative disease susceptibility genetic variant with a complex trait is typically followed by estimation and interpretation of the parameter estimate describing the association. The accuracy of this estimate plays a critical role in the successful design of replication studies and in achieving the goal of reproducibility. Bootstrap bias‐reduction methods provide a general, highly adaptable approach to improve accuracy and guide study design for the conventional approach of one‐at‐a‐time SNP association analysis. The GW bootstrap estimator also shows some promise for utility in the construction of accurate polygenetic risk scores based on single SNP effect estimates and related multiparameter prediction approaches.

## Supporting information

Supplementary Material
