# Supplementary material for: Resampling to Address the Winner's Curse in Genetic Association Analysis of Time to Event
Source: Genet Epidemiol. 2015 Sep 28;39(7):518–28. doi: 10.1002/gepi.21920 (PMC4609263; doi:10.1002/gepi.21920)
Supplement: Supplementary file 1 — Supplementary Material [file GEPI-39-518-s001.pdf]

Additional Results from Simulation Studies

Table S1: Simulation Study 1 Genomewide Bootstrap Summary Statistics (significance threshold *P* <5x10^{-5}) Comparison of the *genomewide bias-reduced bootstrap* and the *naïve* Cox PH estimates for 15 SNPs with MAF ≥ 5% generated to have association with time to severe nephropathy in a sample of 5444 subjects. The rows are ordered by empirical power which is the proportion of simulated datasets in which the SNP was detected as significant out of 5000 replications. Mean bias is calculated as the difference between the mean fitted logHR in all datasets and the mean logHR in selected datasets.

SNP MAF	Data generated logHR	Empirical Power (%)	Mean fitted logHR	Mean values for logHR estimates					
				Uncorrected Naïve	Genome-wide Bootstrap	Bias in Naïve	Bias in Genome-wide	% reduction of Naïve estimate	No. of datasets selected
10.3	0.50	99.5	0.50	0.50	0.43	0.00	-0.07	15.3	4974
34.9	0.19	77.3	0.22	0.23	0.18	0.01	-0.04	24.2	3863
26.7*	0.22	65.5	0.20	0.23	0.17	0.03	-0.03	26.4	3275
17.1	0.17	55.7	0.26	0.30	0.23	0.04	-0.03	24.3	2785
15.0	0.13	52.2	0.27	0.31	0.24	0.04	-0.03	24.2	2611
13.7*	0.23	50.0	0.22	0.26	0.19	0.04	-0.03	29.7	2499
48.8*	0.27	17.2	0.14	0.19	0.14	0.05	0.00	29.2	861
39.0*	0.21	10.0	0.12	0.19	0.13	0.07	0.01	30.8	500
5.8*	0.19	9.3	0.21	0.34	0.23	0.13	0.02	34.0	463
15.8*	0.09	8.9	0.15	0.24	0.16	0.09	0.01	31.6	447
15.8*	0.13	8.4	0.15	0.24	0.16	0.09	0.01	31.0	418
14.6	0.14	0.7	0.12	0.28	0.20	0.16	0.08	28.6	36
36.3	0.14	0.6	0.07	0.19	0.14	0.12	0.07	30.0	29
6.8	0.27	0.2	0.16	0.42	0.31	0.26	0.15	26.9	11
6.9	0.12	0	0.09	NA	NA	NA	NA	NA	0

* minor allele is the risk allele, otherwise major allele is associated with risk

Table S2: Simulation Study 1 Conditional Likelihood Summary Statistics (significance threshold *P* <5x10^{-5}) Comparison of the Ghosh et al *conditional likelihood* and the *naïve* Cox PH estimates for 15 SNPs with MAF ≥ 5% generated to have association with time to severe nephropathy in a sample of 5444 subjects. The rows are ordered by empirical power which is the proportion of simulation datasets in which the SNP was detected as significant out of the 5000 replications. Mean bias is calculated as the difference between the mean fitted logHR in all datasets and the mean logHR in selected datasets.

SNP MAF	Data generated logHR	Empirical Power (%)	Mean fitted logHR	Mean values for logHR estimates					
				Uncorrected Naive	Conditional Likelihood	Bias in Naïve	Bias in Likelihood	% reduction of Naive estimate	No. of datasets selected
10.3	0.50	99.5	0.50	0.50	0.50	0.00	0.00	0.4	4974
34.9	0.19	77.3	0.22	0.23	0.23	0.01	0.01	2.2	3863
26.7*	0.22	65.5	0.20	0.23	0.22	0.03	0.02	2.4	3275
17.1	0.17	55.7	0.26	0.30	0.29	0.04	0.03	3.0	2785
15.0	0.13	52.2	0.27	0.31	0.30	0.04	0.03	3.2	2611
13.7*	0.23	50.0	0.22	0.26	0.25	0.04	0.03	2.9	2499
48.8*	0.27	17.2	0.14	0.19	0.18	0.05	0.04	4.2	861
39.0*	0.21	10.0	0.12	0.19	0.18	0.07	0.06	4.4	500
5.8*	0.19	9.3	0.21	0.34	0.33	0.13	0.12	4.1	463
15.8*	0.09	8.9	0.15	0.24	0.23	0.09	0.08	3.9	447
15.8*	0.13	8.4	0.15	0.24	0.23	0.09	0.08	4.0	418
14.6	0.14	0.7	0.12	0.28	0.27	0.16	0.15	4.9	36
36.3	0.14	0.6	0.07	0.19	0.18	0.12	0.11	4.6	29
6.8	0.27	0.2	0.16	0.42	0.39	0.26	0.23	6.1	11
6.9	0.12	0	0.09	NA	NA	NA	NA	NA	0

* minor allele is the risk allele, otherwise major allele is associated with risk

Table S3: Simulation Study 1 Single-SNP Bootstrap Summary Statistics (significance threshold *P* <5x10^{-5}) Comparison of the *single-SNP bias-reduced bootstrap* and the *naïve* Cox PH estimates for 15 SNPs with MAF ≥ 5% generated to have association with time to severe nephropathy in a sample of 5444 subjects. The rows are ordered by empirical power which is the proportion of simulation datasets in which the SNP was detected as significant out of the 5000 replications. Mean bias is calculated as the difference between the mean fitted logHR in all datasets and the mean logHR in selected datasets.

SNP MAF	Data generated logHR	Empirical Power (%)	Mean fitted logHR	Mean values for logHR estimates					
				Uncorrected Naive	Single SNP Bootstrap	Bias in Naïve	Bias in Single-SNP	% reduction of Naive estimate	No. of datasets selected
10.3	0.50	99.5	0.50	0.50	0.50	0.00	0.00	2.4	4974
34.9	0.19	77.3	0.22	0.23	0.22	0.01	0.00	7.5	3863
26.7*	0.22	65.5	0.20	0.23	0.21	0.03	0.01	9.1	3275
17.1	0.17	55.7	0.26	0.3	0.27	0.04	0.01	9.0	2785
15.0	0.13	52.2	0.27	0.31	0.29	0.04	0.02	9.1	2611
13.7*	0.23	50.0	0.22	0.26	0.24	0.04	0.02	10.2	2499
48.8*	0.27	17.2	0.14	0.19	0.17	0.05	0.03	12.6	861
39.0*	0.21	10.0	0.12	0.19	0.16	0.07	0.04	12.7	500
5.8*	0.19	9.3	0.21	0.34	0.30	0.13	0.09	14.2	463
15.8*	0.09	8.9	0.15	0.24	0.21	0.09	0.06	13.1	447
15.8*	0.13	8.4	0.15	0.24	0.21	0.09	0.06	13.1	418
14.6	0.14	0.7	0.12	0.28	0.25	0.16	0.13	12.6	36
36.3	0.14	0.6	0.07	0.19	0.17	0.12	0.10	13.4	29
6.8	0.27	0.2	0.16	0.42	0.35	0.26	0.19	16.7	11
6.9	0.12	0	0.09	NA	NA	NA	NA	NA	0

* minor allele is the risk allele, otherwise major allele is associated with risk

Table S4: Simulation Study 2 Genomewide Bootstrap Summary Statistics (significance threshold *P* <0.01) Comparison of the *genomewide bias-reduced bootstrap* and the *naïve* Cox PH estimates for 15 SNPs with MAF ≥ 5% generated to have association with time to severe nephropathy in a sample of 5444 subjects. The rows are ordered by empirical power which is the proportion of simulation datasets in which the SNP was detected as significant out of 5000 replications. Mean bias is calculated as the difference between the mean fitted logHR in all datasets and the mean logHR in selected datasets.

SNP MAF	Data generated logHR	Empirical Power (%)	Mean fitted logHR	Mean values for log(HR) estimates					
				Uncorrected Naive	Genome-wide Bootstrap	Bias in Naïve	Bias in Genome -wide	% reduction of Naive estimate	No. of datasets selected
10.3	0.50	100	0.50	0.50	0.42	0.00	-0.08	15.5	5000
34.9	0.19	99.1	0.22	0.22	0.16	0.00	-0.06	27.4	4953
26.7*	0.22	97.6	0.20	0.21	0.15	0.01	-0.05	31.2	4882
17.1	0.17	96.9	0.26	0.26	0.19	0.00	-0.07	29.8	4845
15.0	0.13	96.5	0.27	0.27	0.19	0.00	-0.08	30.2	4824
13.7*	0.23	93.7	0.22	0.23	0.15	0.01	-0.07	36.7	4685
48.8*	0.27	75.4	0.14	0.15	0.09	0.02	-0.04	39.1	3772
39.0*	0.21	66.0	0.12	0.14	0.08	0.02	-0.04	42.2	3300
5.8*	0.09	58.5	0.15	0.18	0.10	0.03	-0.05	44.1	2923
15.8*	0.13	56.7	0.15	0.18	0.10	0.03	-0.05	44.1	2834
15.8*	0.19	54.3	0.21	0.27	0.15	0.06	-0.06	46.7	2714
14.6	0.14	24.0	0.12	0.19	0.11	0.07	-0.01	41.9	1198
36.3	0.14	18.6	0.07	0.13	0.07	0.06	0.0	44.9	929
6.8	0.27	15.1	0.16	0.29	0.17	0.13	0.01	40.1	755
6.9	0.12	4.3	0.09	0.27	0.15	0.18	0.06	42.3	215

* minor allele is the risk allele, otherwise major allele is associated with risk

Table S5: Simulation Study 3 Genomewide Bootstrap Summary Statistics (significance threshold *P* <5x10^{-5}) Comparison of the *genomewide bias-reduced bootstrap* method and the *naïve* Cox PH method for 15 SNPs with MAF ≥ 5% generated to have association with time to severe nephropathy in either direction in a sample of 5444 subjects. The rows are ordered by empirical power which is the number of simulation datasets in which the SNP was detected as significant (p<5x10^{-5}) out of the 5000 replications. Mean bias is calculated as the difference between the mean fitted logHR in all datasets and the mean logHR in selected datasets.

SNP MAF	Data generated logHR	Empirical Power (%)	Mean fitted logHR	Mean values for log(HR) estimates					
				Uncorrected Naive	Genome-wide Bootstrap	Bias in Naïve	Bias in Genome-wide	% reduction of Naive estimate	No. of datasets selected
48.8	0.27	99.8	0.37	0.37	0.33	0.00	-0.04	11.5	4989
10.3*	0.50	98.5	0.45	0.45	0.38	0.00	-0.07	15.7	4924
39.0	0.21	72.0	0.25	0.28	0.22	0.03	-0.03	21.2	3599
13.7*	0.23	59.5	0.29	0.33	0.25	0.04	-0.04	25.2	2977
17.1	0.17	24.4	0.27	0.37	0.29	0.10	0.02	22.2	1219
15.0	0.13	24.0	0.29	0.39	0.31	0.10	0.02	22.0	1199
26.7	0.22	20.6	0.21	0.29	0.22	0.08	0.01	23.9	1031
15.8*	0.09	17.4	0.21	0.30	0.22	0.09	0.01	27.0	870
15.8*	0.13	16.0	0.20	0.30	0.22	0.10	0.02	27.6	799
5.8*	0.19	2.9	0.20	0.41	0.28	0.21	0.08	32.1	147
36.3	0.14	1.0	0.11	0.24	0.18	0.13	0.07	26.3	48
34.9*	0.19	0.1	0.06	0.24	0.18	0.18	0.12	27.4	5
6.8	0.27	0.02	0.09	0.57	0.45	0.48	0.36	20.9	1
14.6	0.14	0.0	0.07	NA	NA	NA	NA	NA	0
6.9	0.12	0.0	0.04	NA	NA	NA	NA	NA	0

* minor allele is the risk allele, otherwise major allele is associated with risk

Supplemental Figures:

Figure S3: Simulation Study 1 Conditional Likelihood Estimates for True Positive SNPs (significance threshold $P < 5x10^{-5}$)

Figure S4: Simulation Study 1 Conditional Likelihood Estimates for False Positive SNPs (significance threshold $P < 5x10^{-5}$)

Figure S5: Simulation Study 1 Genomewide Bootstrap and Conditional Likelihood Estimates for True Positive SNPs (significance threshold $P < 5x10^{-5}$)

Figure S6: Simulation Study 1 Genomewide Bootstrap and Conditional Likelihood Estimates for False Positive SNPs (significance threshold $P < 5x10^{-5}$)

Figure S7: Simulation Study 1 Single-SNP Bootstrap Estimates for True Positive SNPs (significant threshold $P < 5x10^{-5}$)

Figure S8: Simulation Study 1 Genomewide Bootstrap and Single-SNP Bootstrap Estimates for True Positive SNPs (significance threshold $P < 5x10^{-5}$)

Figure S9: Simulation Study 2 Genomewide Bootstrap Estimates for True Positive SNPs (significance threshold $P < 0.01$)

Figure S10: Simulation Study 2 Genomewide Bootstrap Estimates for False Positive SNPs (significance threshold $P < 0.01$)

Figure S11: Simulation Study 3 Genomewide Bootstrap Estimates for True Positive SNPs (significance threshold $P < 5x10^{-5}$)

Figure S12: Simulation Study 3 Genomewide Bootstrap Estimates for False Positive SNPs (significance threshold $P < 5x10^{-5}$)

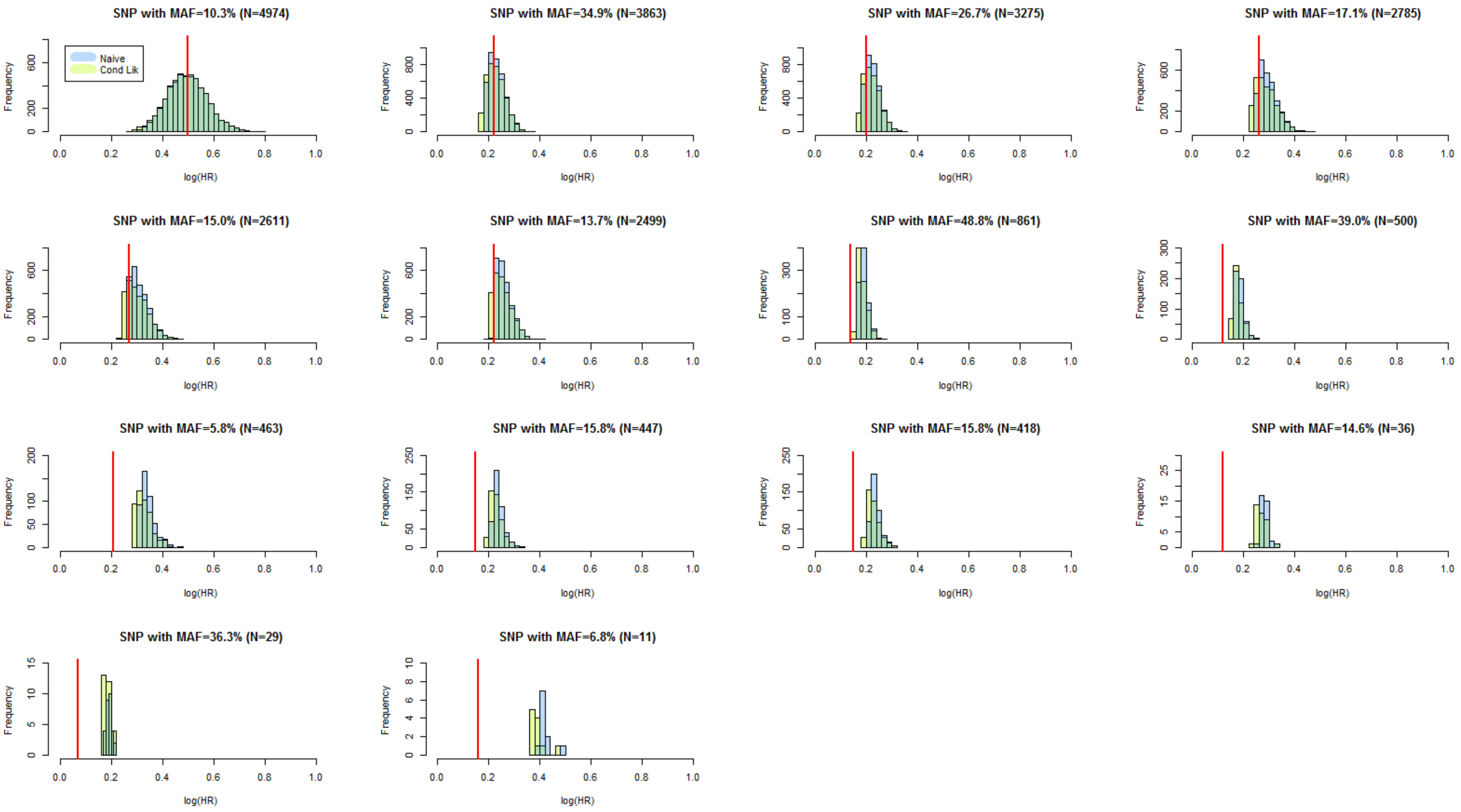

Figure S3: Simulation Study 1 Conditional Likelihood Estimates for True Positive SNPs (significance threshold *P* <5x10^{-5}) Comparison of distributions of *conditional likelihood* (transparent yellow Cond Lik) and uncorrected *naive* (transparent blue) logHR estimates of true positive SNPs with MAF ≥ 5% out of the 5000 replications of a sample of 5444 subjects. The vertical solid red line denotes the fitted logHR averaged across unselected datasets. The SNPs are ordered by number of simulation datasets (N) in which the SNP was detected as statistically significant (see Table S2).

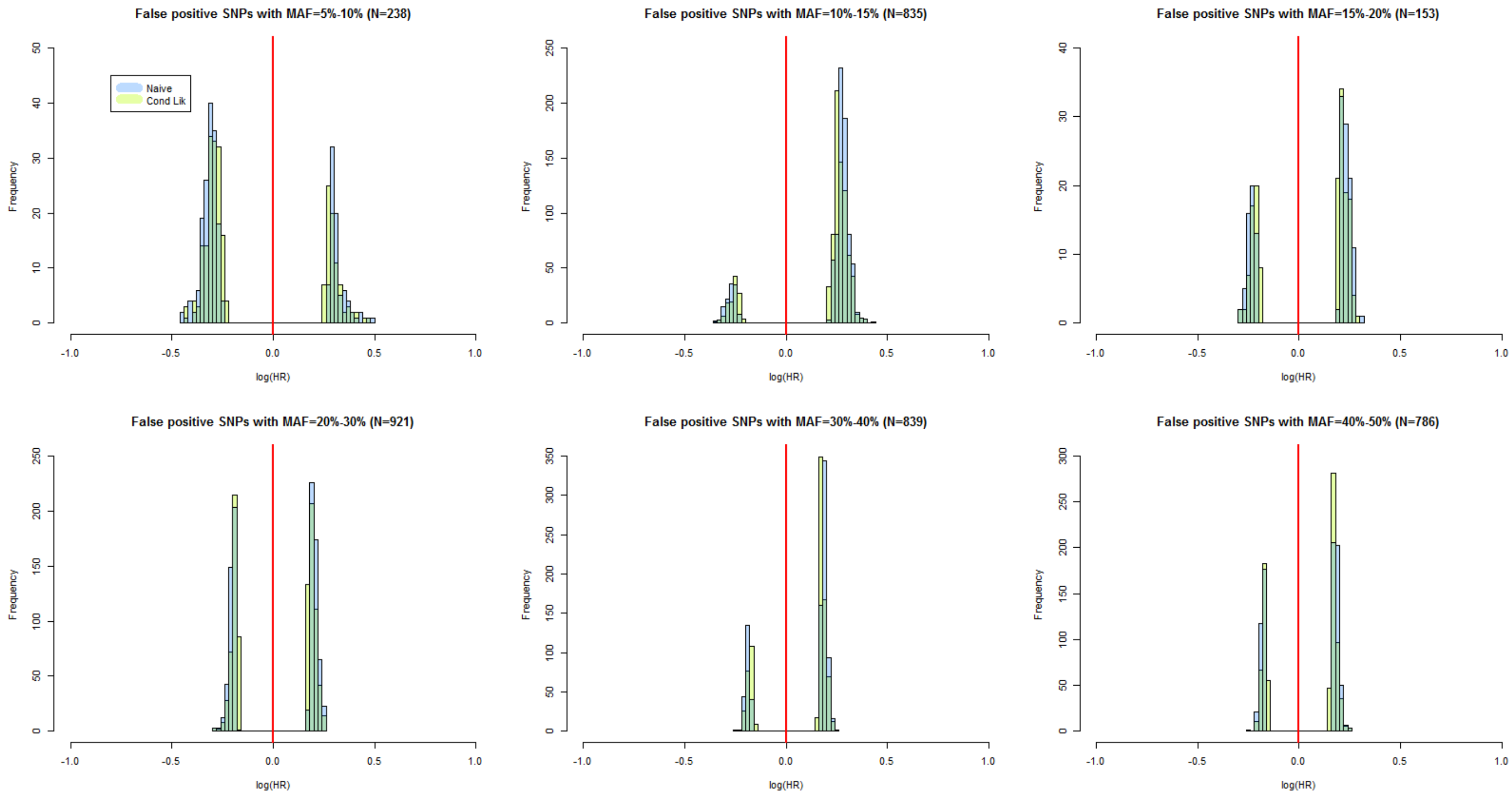

Figure S4: Simulation Study 1 Conditional Likelihood Estimates for False Positive SNPs (significance threshold $P < 5\times10^{-5}$)
Comparison of distributions of *conditional likelihood* (transparent yellow Cond Lik) and uncorrected *naive* (transparent blue) logHR estimates of false positive SNPs among 5000 replications of a sample of 5444 subjects, stratified by MAF categories. False positive SNPs are those found to be statistically significant and not in the same gene as any of the SNPs in the model used for data generation. The vertical solid red line denotes the null reference value.

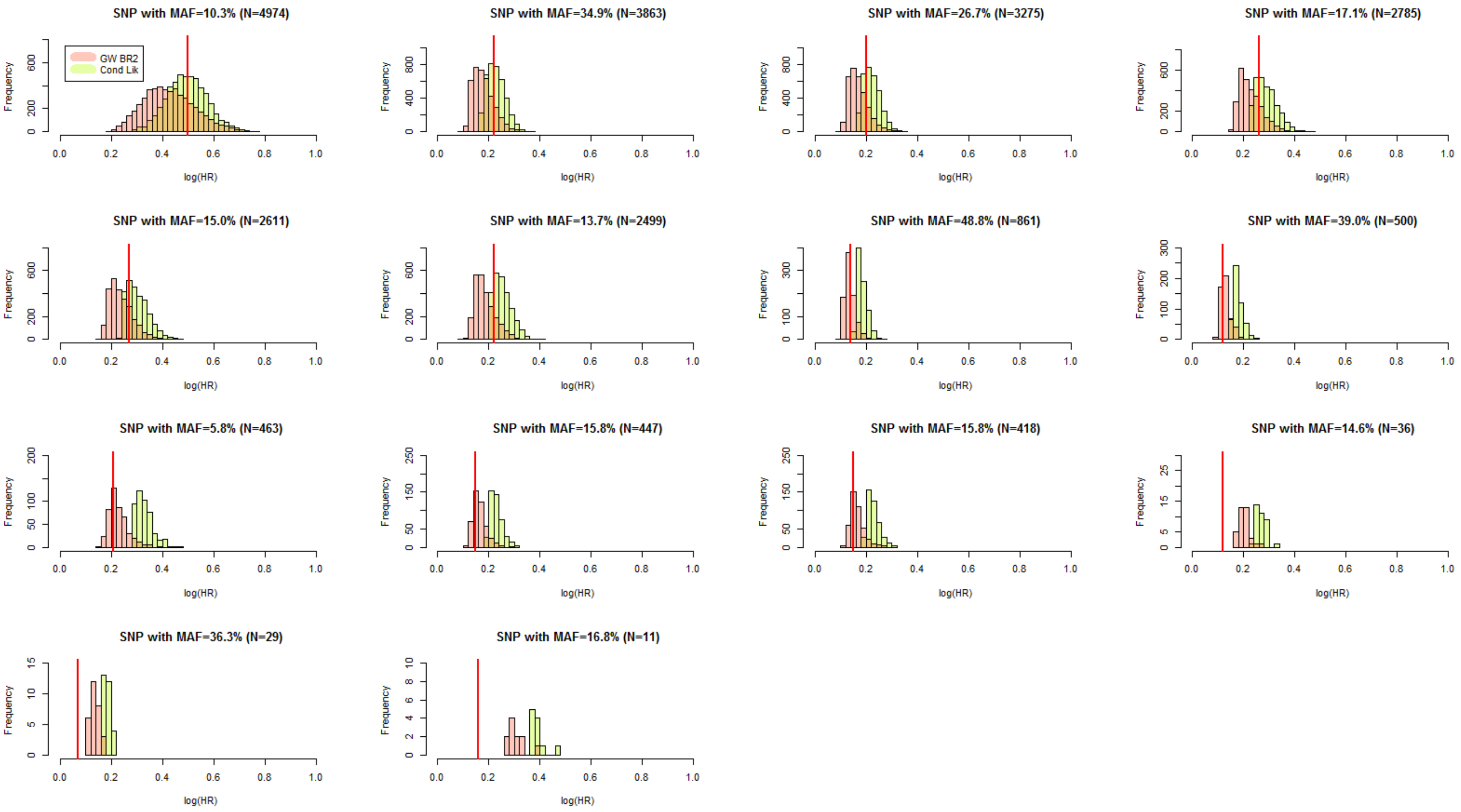

Figure S5: Simulation Study 1 Genomewide Bootstrap and Conditional Likelihood Estimates for True Positive SNPs (significance threshold *P* <5x10^{-5}) Comparison of distributions of *genome-wide bootstrap* (transparent red GW BR2) and *conditional likelihood* (transparent yellow Cond Lik) logHR estimates of true positive SNPs with MAF ≥ 5% out of the 5000 replications of a sample of 5444 subjects. The vertical solid red line denotes the fitted logHR averaged across unselected datasets. The SNPs are ordered by number of simulation datasets (N) in which the SNP was detected as statistically significant (see Tables 2, S1, S2).

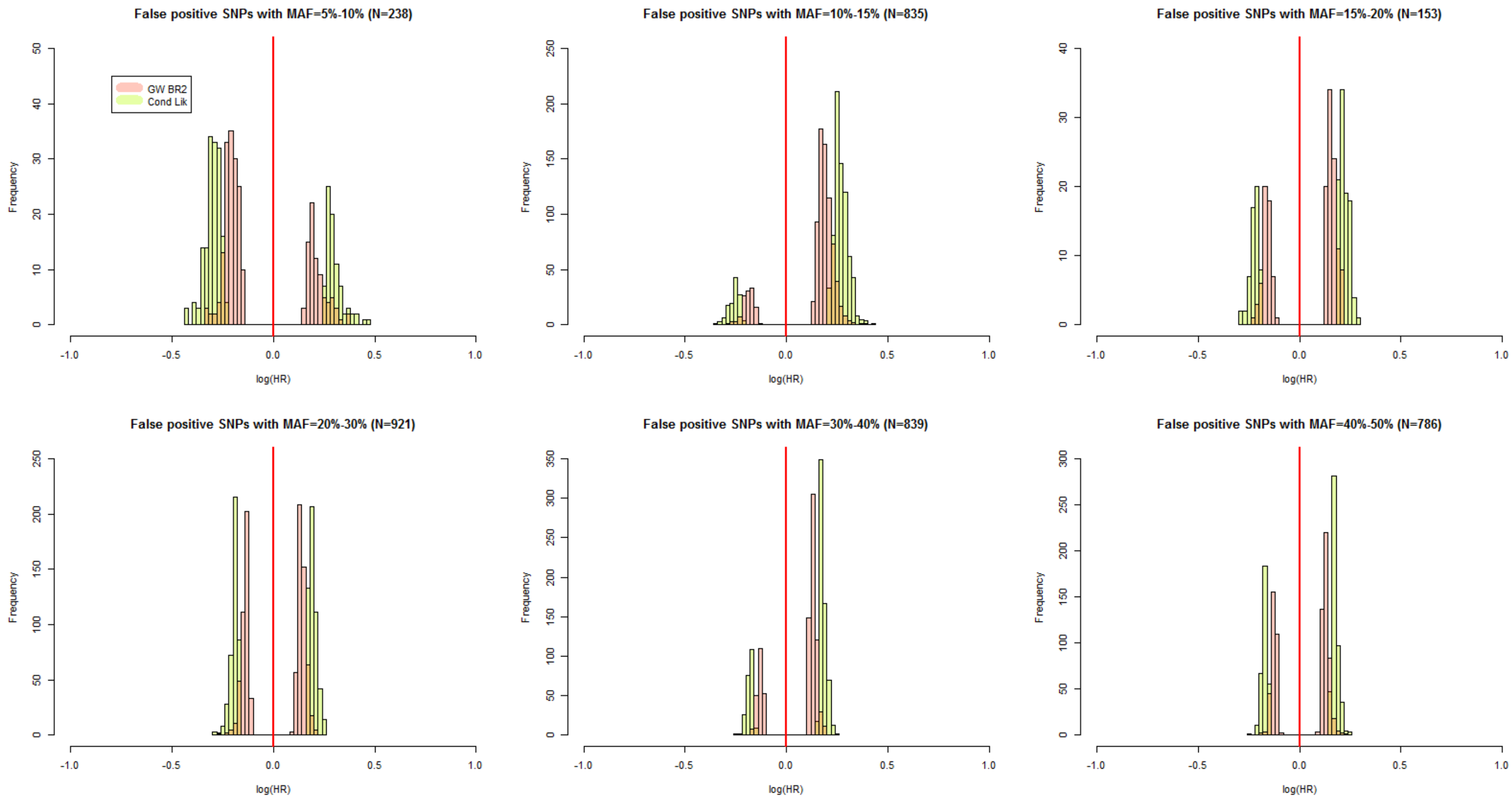

Figure S6: Simulation Study 1 Genomewide Bootstrap and Conditional Likelihood Estimates for False Positive SNPs (significance threshold $P < 5x10^{-5}$) Comparison of distributions of *genome-wide bootstrap* (transparent red GW BR2) and *conditional likelihood* (transparent yellow Cond Lik) logHR estimates of false positive SNPs among 5000 replications of a sample of 5444 subjects, stratified by MAF categories. False positive SNPs are those found to be statistically significant and not in the same gene as any of the SNPs in the model used for data generation. The vertical solid red line denotes the null reference value.

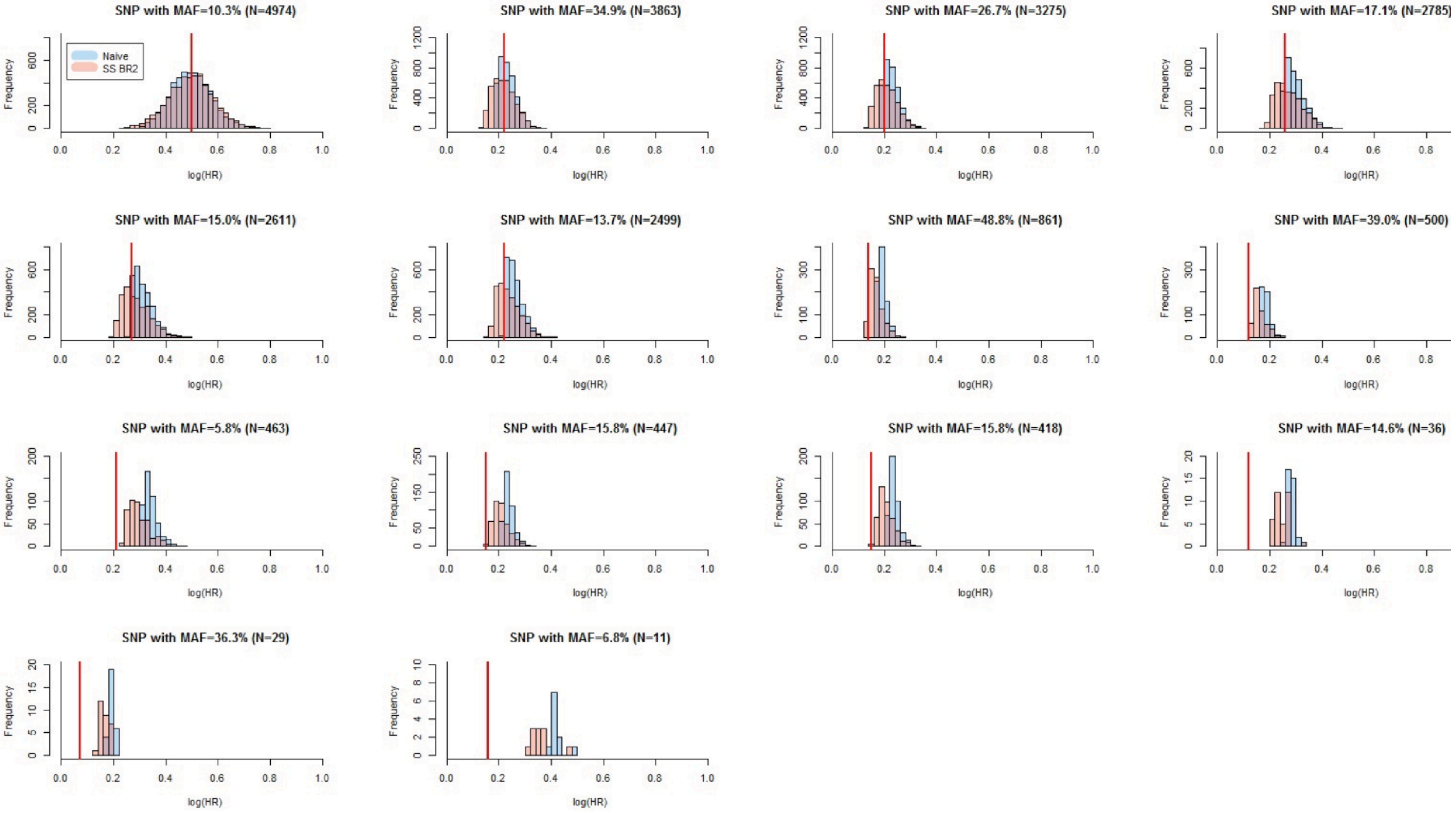

Figure S7: Simulation Study 1 Single-SNP Bootstrap Estimates for True Positive SNPs (significant threshold *P* <5x10^{-5}). Comparison of distributions of *single-SNP bootstrap* (transparent red SS BR2) and uncorrected *naive* (transparent blue) logHR estimates of true positive SNPs with MAF ≥ 5% out of 5000 replications in a sample of 5444 subjects. The vertical solid red line denotes the fitted logHR averaged across unselected datasets. The SNPs are ordered by number of simulation datasets (N) in which the SNP was detected as statistically significant (see Table 2).

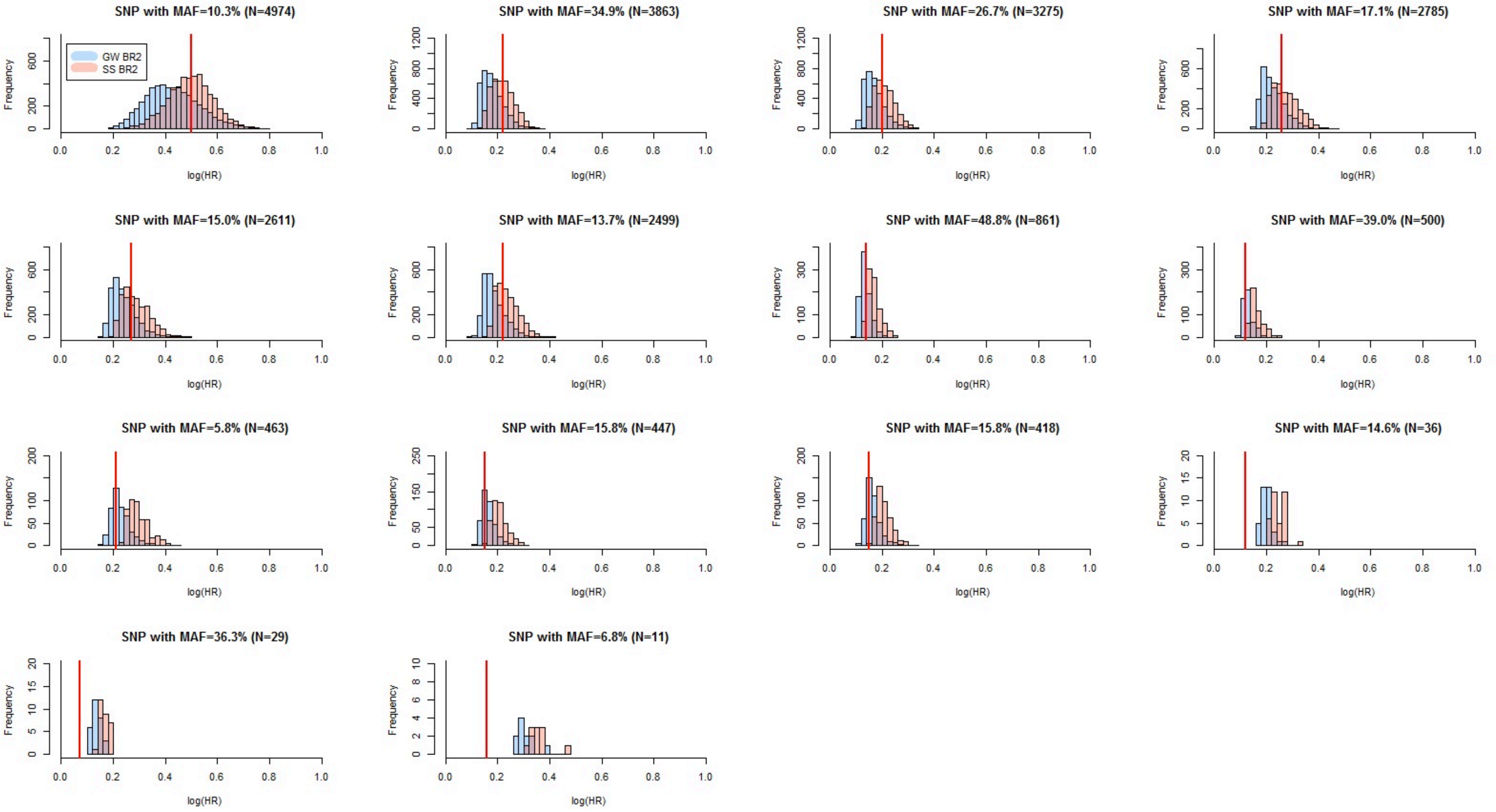

Figure S8: Simulation Study 1 Genomewide Bootstrap and Single-SNP Bootstrap Estimates for True Positive SNPs (significance threshold *P* <5x10^{-5}) Comparison of distributions of *genome-wide bootstrap* (transparent blue MS BR2) and *single-SNP bootstrap* (transparent red SS BR2) logHR estimates of true positive SNPs with MAF ≥ 5% out of 5000 replications of a sample of 5444 subjects. The vertical solid red line denotes the fitted logHR averaged across unselected datasets. The SNPs are ordered by number of simulation datasets (N) in which the SNP was detected as statistically significant (see Tables 2, S1, S3).

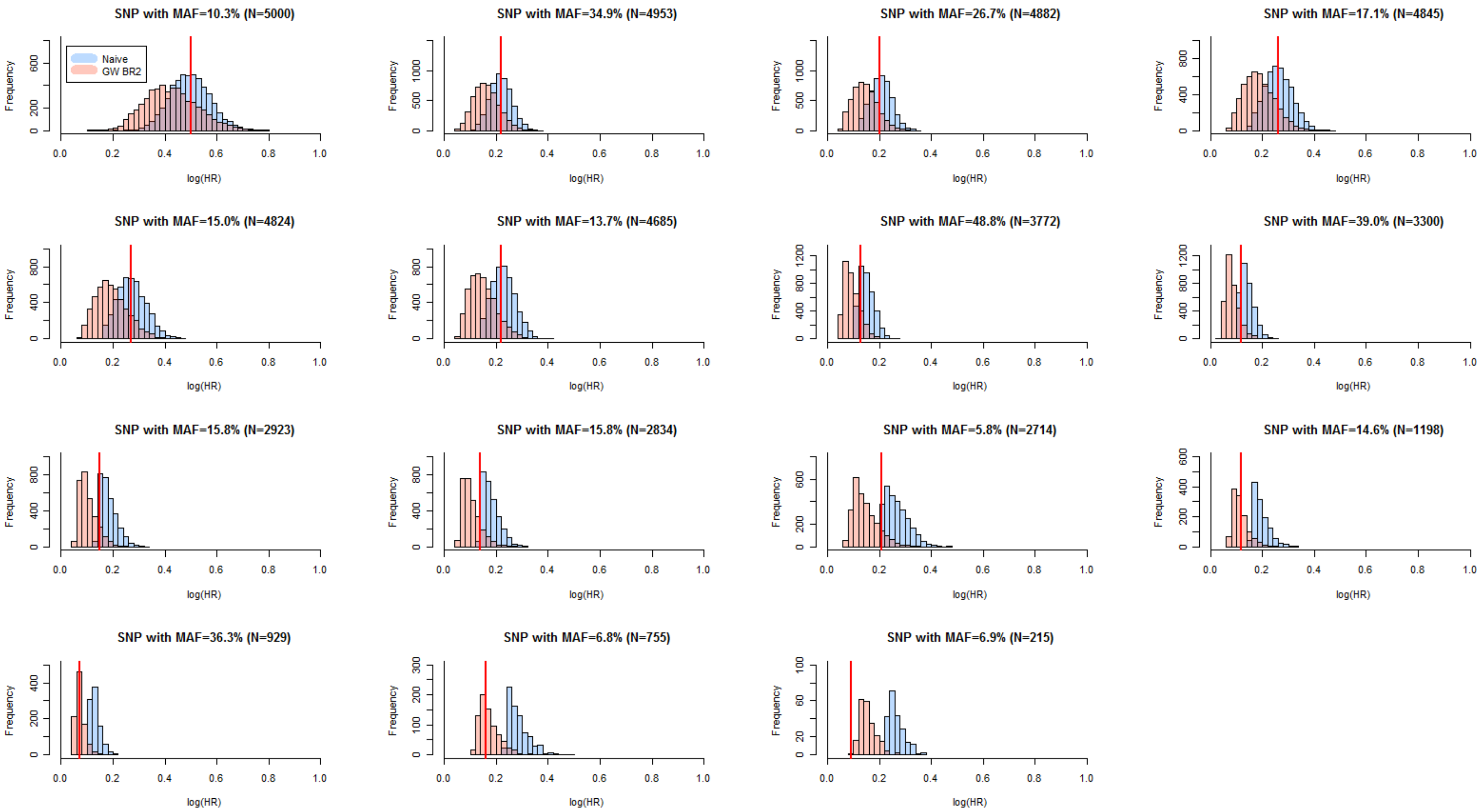

Figure S9: Simulation Study 2 Genomewide Bootstrap Estimates for True Positive SNPs (significance threshold *P* <0.01) Comparison of distributions of *genome-wide bootstrap* (transparent red GW BR2) and uncorrected *naive* (transparent blue) logHR estimates of true positive SNPs with MAF ≥ 5% out of the 5000 replications of a sample of 5444 subjects. The vertical, solid red line denotes the fitted logHR averaged across unselected datasets. The SNPs are ordered by number of simulation datasets (N) in which the SNP was detected as statistically significant (see Table S4).

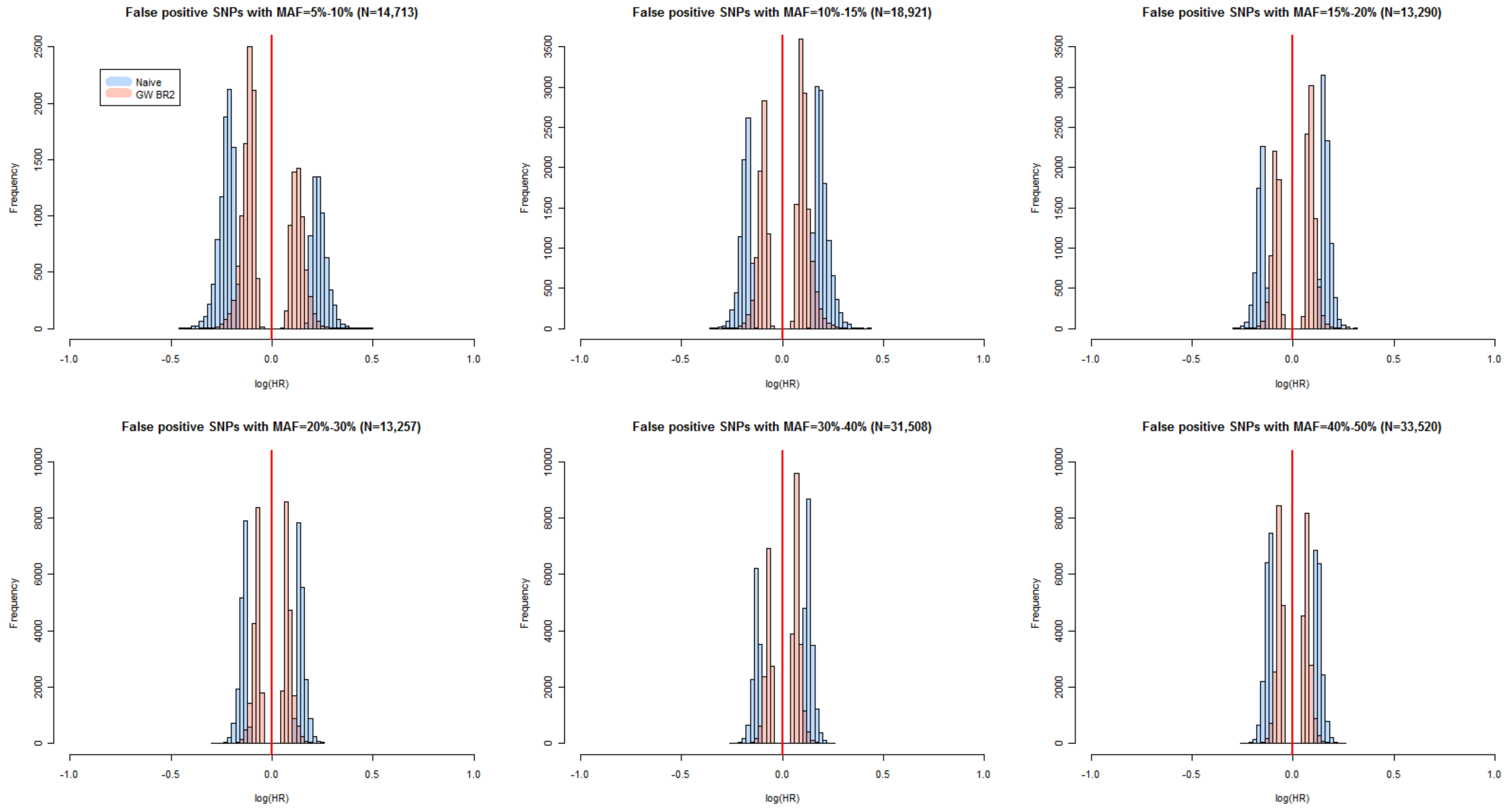

Figure S10: Simulation Study 2 Genomewide Bootstrap Estimates for False Positive SNPs (significance threshold *P* <0.01)
Comparison of distributions of *genome-wide bootstrap* (transparent red GW BR2) and uncorrected *naive* (transparent blue) logHR estimates of false positive SNPs in a sample of 5444 subjects, stratified by MAF categories. False positive SNPs are those found to be statistically significant among 5000 replications and not in the same gene as any of the SNPs in the model used for data generation. The vertical solid red line denotes the null reference value.

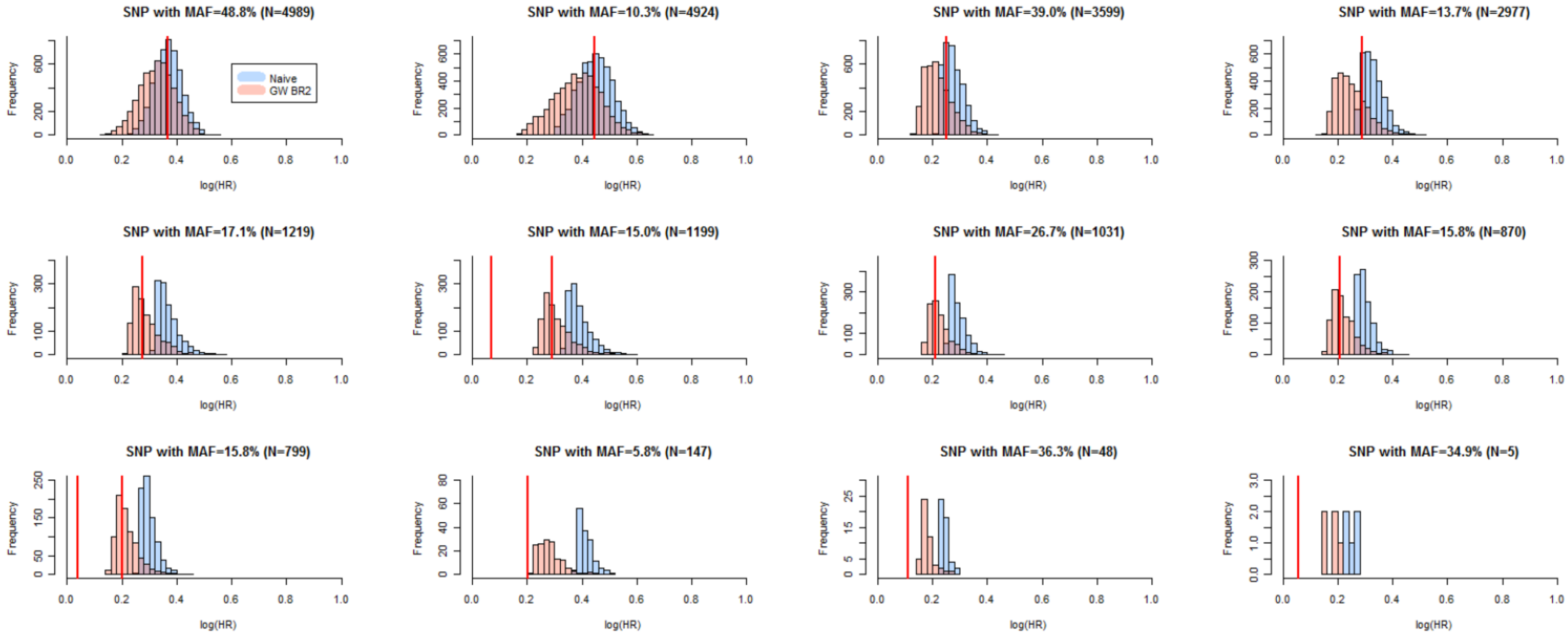

Figure S11: Simulation Study 3 Genomewide Bootstrap Estimates for True Positive SNPs (significance threshold *P* <5x10^{-5})
Comparison of distributions of *genome-wide bootstrap* (transparent red GW BR2) and uncorrected *naive* (transparent blue) logHR estimates of true positive SNPs with MAF ≥ 5% out of the 5000 replications of a sample of 5444 subjects. The vertical, solid red line denotes the fitted logHR averaged across unselected datasets. The SNPs are ordered by number of simulation datasets (N) in which the SNP was detected as statistically significant (see Table S5).

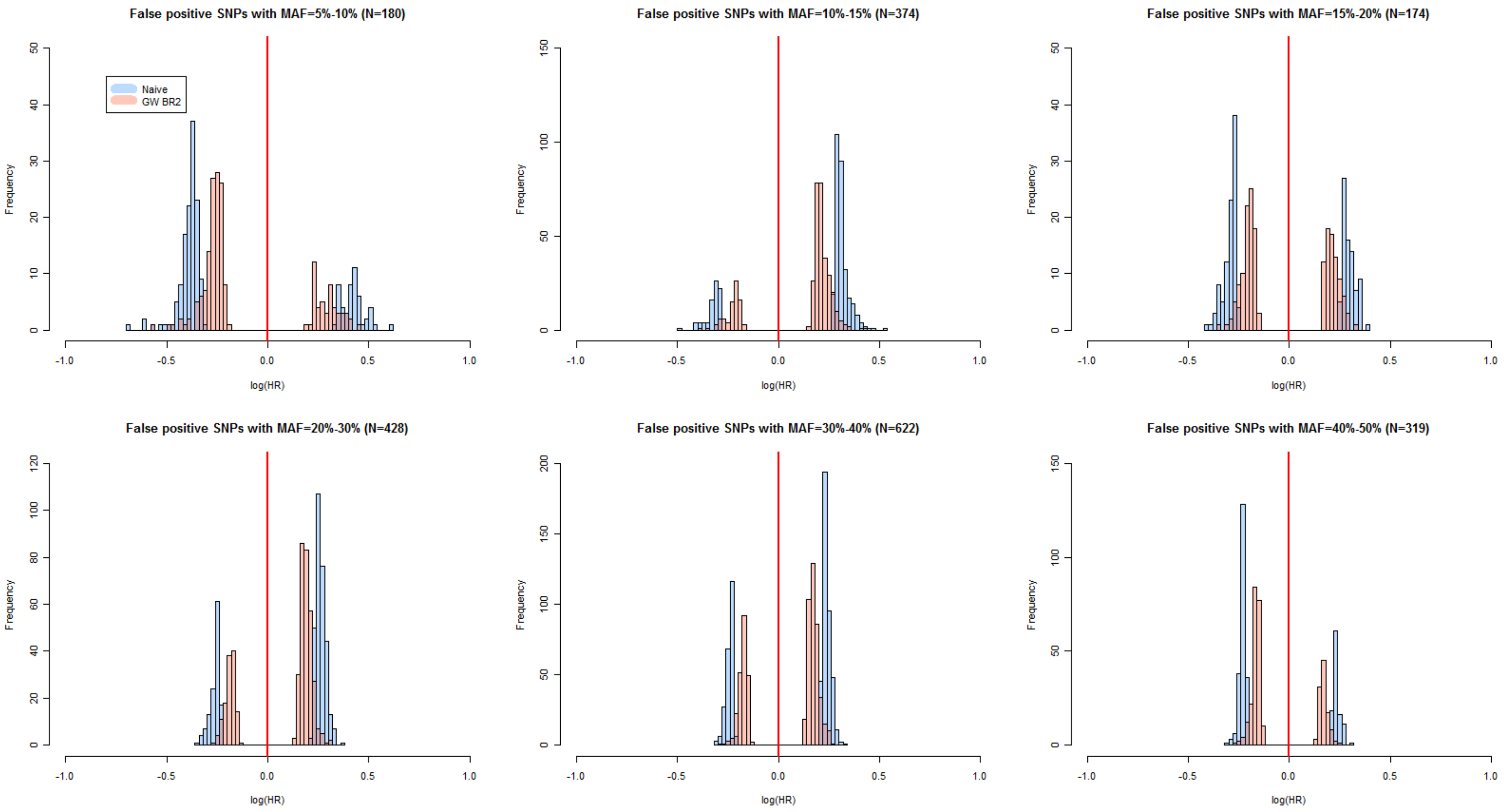

Figure S12: Simulation Study 3 Genomewide Bootstrap Estimates for False Positive SNPs (significance threshold $P < 5x10^{-5}$) Comparison of distributions of *genome-wide bootstrap* (transparent red GW BR2) and uncorrected *naive* (transparent blue) logHR estimates of false positive SNPs in a sample of 5444, stratified by MAF categories. False positive SNPs are those found to be statistically significant among 5000 replications and not in the same gene as any of the SNPs in the model used for data generation. The vertical solid red line denotes the null reference value.
